# A key role of the WEE1-CDK1 axis in mediating TKI-therapy resistance in FLT3-ITD positive acute myeloid leukemia patients

**DOI:** 10.1038/s41375-022-01785-w

**Published:** 2022-12-12

**Authors:** Giorgia Massacci, Veronica Venafra, Sara Latini, Valeria Bica, Giusj Monia Pugliese, Simone Graziosi, Felix Klingelhuber, Natalie Krahmer, Thomas Fischer, Dimitrios Mougiakakos, Martin Boettcher, Livia Perfetto, Francesca Sacco

**Affiliations:** 1 PhD Program in Cellular and Molecular Biology, Department of Biology, University of Rome “Tor Vergata”, Rome, Italy; 2 Department of Biology, University of Rome Tor Vergata, Via della Ricerca Scientifica 1, 00133 Rome, Italy; 3 Institute for Diabetes and Obesity, Helmholtz Zentrum München, German Center for Diabetes Research, 85764 Neuherberg, Germany; 4 Institute of Molecular and Clinical Immunology, University of Magdeburg, Magdeburg, Germany; 5 Healthcampus for Inflammation, Immunity and Infection (GCI3), University of Magdeburg, Magdeburg, Germany; 6 Department of Hematology and Oncology, University of Magdeburg, Magdeburg, Germany; 7 Department of Biology, Fondazione Human Technopole, Via Rita Levi-Montalcini 1, 20157 Milan, Italy; 8 Telethon Institute of Genetics and Medicine (TIGEM), Via Campi Flegrei 34, Pozzuoli, 80078 Italy

**Keywords:** Cancer, Acute myeloid leukaemia

## Abstract

The insertion site of the internal tandem duplications (ITDs) in the FLT3 gene affects the sensitivity to tyrosine kinase inhibitors (TKIs) therapy in acute myeloid leukemia (AML). Patients with the ITD in the tyrosine kinase domain lack effective therapeutic options. Here, to identify genotype-driven strategies increasing the TKI therapy efficacy, we developed *SignalingProfiler*, a strategy supporting the integration of high-sensitive mass spectrometry-based (phospho)proteomics, RNA sequencing datasets with literature-derived signaling networks. The approach generated FLT3-ITD genotype-specific predictive models and revealed a conserved role of the WEE1-CDK1 axis in TKIs resistance. Remarkably, pharmacological inhibition of the WEE1 kinase synergizes and strengthens the pro-apoptotic effect of TKIs therapy in cell lines and patient-derived primary blasts. Finally, we propose a new molecular mechanism of TKIs resistance in AML and suggest the combination of WEE1 inhibitor and TKI as a therapeutic option to improve patients clinical outcome.

## Introduction

Internal tandem duplications (ITDs) of the FLT3 gene are observed in about 25% of young adults with newly diagnosed acute myeloid leukemia (AML) [1, 2]. The FLT3 gene encodes a receptor tyrosine kinase, consisting of an extracellular immunolike-domain, a transmembrane region, a cytoplasmic juxtamembrane domain (JMD) followed by two tyrosine kinase domains (TKD1 and TKD2) [3]. FLT3-ITD mutations always occur in exons 15 and 16, encoding the JMD and TKD1 regions, and cause its constitutive activation [4]. In 2017, the CALGB 10603/RATIFY trial demonstrated a significantly improved outcome in a cohort of 717 patients carrying genetic alterations in the FLT3 gene when treated with the multikinase inhibitor midostaurin (PKC412) combined with standard frontline chemotherapy [5]. At the beginning of 2022, a retrospective analysis of the same trial evaluated role of different insertion sites of ITD mutations in predicting response to midostaurin treatment. Interestingly, the analysis revealed that midostaurin treatment exerted a significant beneficial effect only in patients carrying the ITDs in the JMD domain, whereas no beneficial effect was observed in patients carrying ITDs in the TKD region. In addition, multivariate analysis showed that the ITD-TKD localization is an unfavorable prognostic factor for overall survival and incidence of relapse [6].

In accordance with these clinical observations, previous in vitro studies showed that ITDs-TKD confer resistance to chemotherapy and are associated with a significantly worse outcome. Briefly, ITD-TKD positive cell lines and primary mouse bone marrow cells showed reduced apoptosis when compared to ITDs-JMD, upon exposure to FLT3 inhibitors, namely midostaurin and quizartinib (a highly-specific second-generation FLT3 inhibitor) [7–9].

Interestingly, the enzymatic activity of ITD-TKD and ITD-JMD is equally suppressed by kinase inhibitors[7], suggesting that the different TKI sensitivity may be caused by an extensive rewiring of cell signaling network. We, therefore, performed a system-level analysis of the state of FLT3-ITD cells induced by TKIs treatment. To obtain FLT3-ITD cell-specific models, we developed “*SignalingProfiler*”, a novel, generally applicable, computational strategy supporting the integration of these large “omic” datasets with literature-derived causal networks. This strategy highlighted the novel and crucial role of the WEE1-CDK1 axis in TKI therapy failure in FLT3^ITD-TKD^ patients. Remarkably, pharmacological inhibition of WEE1 completely rescued the ability of patient-derived primary blasts, carrying the ITD-TKD mutation to undergo apoptosis in response to midostaurin treatment. Our strategy is generally applicable to the study of drug resistance and mechanism of action, and can lead to the identification of novel therapeutic targets for combination therapy.

## Materials and methods

Cell lines, MS-based (phospho)proteomic and deep sequencing workflow, bioinformatic and statistical data analysis, cell viability assay, western blotting, flow cytometry analyses and patient-derived primary blasts experiments are described in Supplementary Materials and Methods section.

### Apoptosis assay

Cells were plated at a concentration of 500.00 cells/ml and treated as indicated. After incubation for 24 h, apoptotic cells were measured by flow cytometry using Ebioscience™ Annexin V Apoptosis Detection Kit APC according to the kit instruction (Cat. 88-8007-74, Thermo Fisher Scientific). Cells positive for annexin-V were counted as apoptotic cells.

## Results

### The experimental strategy

**Fig. 1:**
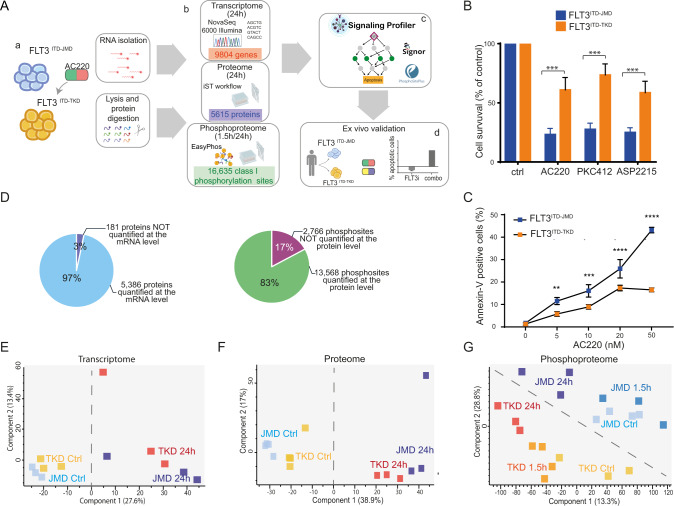
The different ITD location affects the sensitivity to TKIs therapy modulating the phosphoproteome of FLT3-ITD cell lines. **A** Overview of the experimental and bioinformatic strategy. BaF3 cells expressing FLT3^ITD-TKD^ (in orange) and FLT3^ITD-JMD^ (in blue) were treated with 20 nM quizartinib (AC220) for 24 h (a). mRNAs were isolated for the transcriptome analysis and the protein extracts were digested and characterized at the proteome and phosphoproteome levels (b). Multi-omics profiles of FLT3-ITD cells were used in *SignalingProfiler* pipeline to obtain cell-specific models and to identify additional druggable genes (c). Proteins of interest were further investigated through complementary assays in patients-derived primary blasts (d). **B** Cell survival of BaF3 cells expressing FLT3^ITD-JMD^ (in blue) and FLT3^ITD-TKD^ (in orange) after FLT3 inhibitors treatment. Cells were treated for 24 h with 20 nM quizartinib (AC220), 100 nM midostaurin (PKC412) and 50 nM gilteritinib (ASP2215). Cell viability was assessed by MTT assay. **C** Induction of apoptosis in BaF3 cells expressing FLT3^ITD-JMD^ (in blue) and FLT3^ITD-TKD^ (in orange) treated with increasing doses of AC220 for 24 h. The percentage of apoptotic cells was determined by Annexin-V labeling. **D** Pie charts representing the percentage and the number of species characterized at the protein and the transcript levels (top) or at the protein and the phosphosite levels (bottom). **E**–**G** Principal Component Analysis (PCA) of the analytes quantified across the transcriptome (**E**), proteome (**F**) and phosphoproteome (**G**) replicates.

Here, we devised a multi-step strategy that combines system-level and unbiased multi-omic analyses (Fig. 1A panel a and b) with literature-derived causal networks to generate cell-specific models (Fig. 1A panel c). We demonstrate that these models have a translational impact and can be used as a framework to identify and test novel drug targets abrogating TKI resistance (Fig. 1A panel d).

Ba/F3 cells stably expressing the FLT3 gene with ITD insertions in the JMD (aa 598) or in the TKD1 (aa 613) region (henceforth “FLT3^ITD-JMD^” and “FLT3^ITD-TKD^” cells, respectively) were treated with FLT3 inhibitors and cells viability and apoptosis were tested. In agreement with previously published data [7, 8], nanomolar concentrations (20–100 nM) of TKIs induced apoptosis in Ba/F3-cells harboring ITD-JMD mutation in a dose dependent manner, whereas ITD-TKD cells showed significantly decreased sensitivity to the three inhibitors (Fig. 1B, C)

### Deep transcriptome, proteome and phosphoproteome analysis of quizartinib treated FLT3-ITD cells

To investigate the molecular basis of the observed different sensitivities to treatment with FLT3 inhibitors in ITD-TKD and ITD-JMD expressing cells, we applied an unbiased strategy to monitor the transcriptional, translational, and post-translational changes induced by FLT3 inhibition. In these large-scale experiments, FLT3-ITD cells were exposed to short (1.5 h) or long (24 h)-term treatments with 20 nM quizartinib (Fig. 1A panel b) to capture the TKI-dependent early and late signaling events at two time points relevant for patient treatment [10]. Among the FLT3 inhibitors, we selected quizartinib, because of its high specificity, and we treated cells for 24 h with 20 nM, a non-toxic concentration whereby the quizartinib-induced apoptotic response is significantly different between FLT3-ITD cells (Fig. 1C).

RNA sequencing approach and state-of-the-art mass spectrometry (MS)-based (phospho)proteomic enabled the quantification of the expression of more than 11,000 genes (Table S1), more than 5000 proteins (Table S2) and 16,000 phosphorylation events (class I sites, Table S3) (Fig. S1A–C). The biological triplicates or quadruplicates were highly correlated with Pearson correlation coefficients ranging between 0.85 (for phospho measurements) and 0.97 (for transcriptome and proteome measurements) (Fig. S1D–F).

The experimental system appeared to be efficient: for nearly all the quantified proteins (97%), we also obtained the levels of the corresponding transcripts (Fig. 1D top panel). Similarly, for approximately 83% of the quantified phosphorylation sites, we also measured protein abundance (Fig. 1D bottom panel). Quizartinib-induced changes at the transcript level tended to correlate with those at the proteome level in FLT3^ITD-JMD^ and FLT3^ITD-TKD^ cells (PC = 0.6–0.7) (Fig. S2A, B). Finally, after normalizing by the protein levels, more than 70% of phosphosites were still significantly regulated by quizartinib treatment (Fig. S2C, D).

Next, we applied a statistical t-test to narrow-down the species that are regulated by the FLT3 inhibitor. Briefly, about one third of the transcriptome, proteome and phosphoproteome displayed a significant (FDR < 0.1) change in the abundance upon quizartinib treatment (Fig. S3A–C). Comparative analysis of significantly modulated genes, proteins or phosphosites, revealed a common core (14% in the transcriptomics, 7% in the proteomics and 5% in the phosphoproteomics) of canonical FLT3-ITD targets significantly altered by 24 h quizartinib treatment, regardless of the ITD insertion site (Fig. S3A–C). Interestingly, our data suggest that the two different ITD localization impacts the quizartinib-dependent remodeling of the phosphoproteome profile to a greater extent as compared to the transcriptome and proteome profile (R phosphoproteome = 0.58) (Fig. S3D–F).

Consistently, principal component analysis (PCA) clearly showed that only the phosphoproteome information better stratify cells according to both FLT3 activation status and ITD insertion site (Fig. 1E–G). Unsupervised hierarchical clustering of our large-scale datasets confirmed that the phosphoproteomic profile best discriminates FLT3 cells according to their quizartinib sensitivity (Fig. S3G–I). These observations indicate that the different localization of the ITD mutations mostly impact the cell regulatory network at the post-translational level, which may drive the different sensitivity to TKI-therapy.

### Pathway modulation in response to quizartinib treatment

**Fig. 2:**
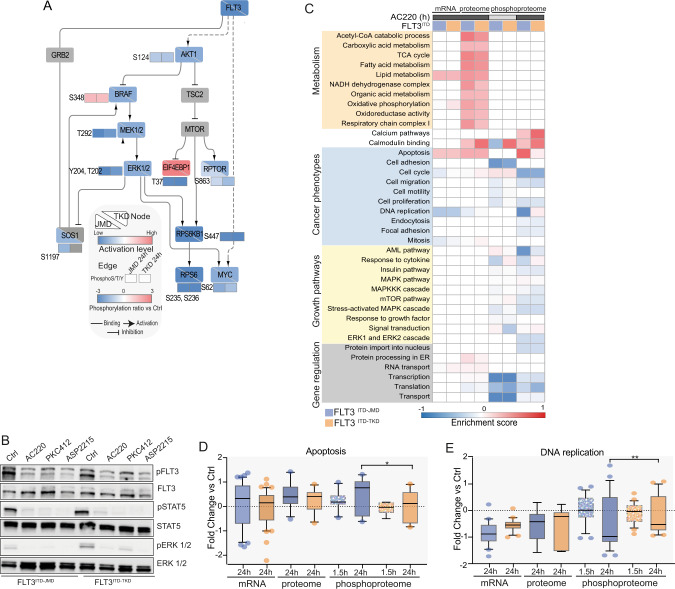
AC220-induced remodelling of the proteome and the phosphoproteome of FLT3^ITD-JMD^ and FLT3^ITD-TKD^ cells. **A** FLT3 downstream causal interaction network. The effect of quizartinib (AC220) on the phosphoproteome and proteome profiles of FLT3^ITD-JMD^ and FLT3^ITD-TKD^ cells was mapped on a literature curated signaling network, extracted from the SIGNOR resource [11]. For comparative analysis, for each node, the activation state in both cell line is shown (down-left half for ITD-JMD, and top-right half for ITD-TKD). Activated proteins are marked in red, whereas inhibited ones in blue. Phosphosites are displayed as independent rectangles and are colored according to their phosphorylation state after quizartinib treatment, as indicated in the legend. **B** Representative western blot showing the inhibition of canonical FLT3 downstream targets, as revealed by their phosphorylation status: Tyr694 in STAT5 and Thr202 and Tyr204 in ERK1/2. ITD-JMD and ITD-TKD BaF3 cells were treated for 1.5 h with FLT3 inhibitors treatment (AC220: quizartinib, PKC412: midostaurin and ASP2215: gilteritinib). **C** Heatmap displaying the enrichment score of GO Biological processes and KEGG pathways significantly (FDR < 0.05) over- (red color) or under- (blue color) represented in the relative dataset (transcripts, proteins and phosphosites in FLT3^ITD-JMD^ (in blue) and FLT3^ITD-TKD^ (in orange) BaF3 cells upon quizartinib (AC220) treatment. **D**, **E** Boxplots showing the relative abundance of significantly modulated transcripts, proteins and phosphoproteins involved in apoptosis (**D**) or DNA replication process (**E**) in BaF3 cells expressing FLT3^ITD-JMD^ (in blue) and FLT3^ITD-TKD^ (in orange) upon quizartinib (AC220) treatment.

We next assessed the effect of quizartinib treatment on previously identified signaling pathways downstream of FLT3. By overlaying our phosphoproteomic results with the FLT3 subnetwork, retrieved from the signaling database SIGNOR [11], we observed that the MAPK and AKT-mTOR pathways are equally inhibited by either short-term and long-term exposure to quizartinib in both cell lines (Fig. 2A). Consistently with previous reports [9], the phosphorylation level of the FLT3-ITD mutants as well as their down-stream canonical targets are equally decreased by TKIs treatments (Fig. 2B).

Next, we checked for biological processes altered in treated cells (as described in Matherials and methods). Gene ontology term enrichment analysis revealed a significant overexpression of proteins involved in mitochondrial metabolic processes, such as TCA cycle and OxPhos, as well as in lipid oxidation (Fig. 2C and Fig. S4A–E).

Consistently with the different sensitivity of FLT3^ITD-JMD^ and FLT3^ITD-TKD^ cells to quizartinib treatment, we found that proteins involved in apoptosis were significantly hyperphosphorylated in FLT3^ITD-JMD^ cells, but not in FLT3^ITD-TKD^ cells (Fig. 2D). Interestingly, we observed that the quizartinib-dependent phosphorylation of DNA replication proteins is significantly decreased only in quizartinib treated FLT3^ITD-JMD^ cells, but not in cells with the ITD mutation in the TKD region (Fig. 2E).

Kinase substrate motifs analysis showed that pro-proliferative kinases, ERK1/2, AKT, and p70S6K are significantly downregulated (FDR < 0.05), in line with the anti-proliferative effect of quizartinib in FLT3-ITD cells (Fig. S4F).

These observations provide a global picture of the main changes induced by quizartinib treatment at the transcriptome, proteome, and phosphoproteome level in both FLT3-ITD cells, but do not clarify the molecular mechanisms underlying their different sensitivity to quizartinib treatment.

### From FLT3 to transcription factors through *SignalingProfiler*

**Fig. 3:**
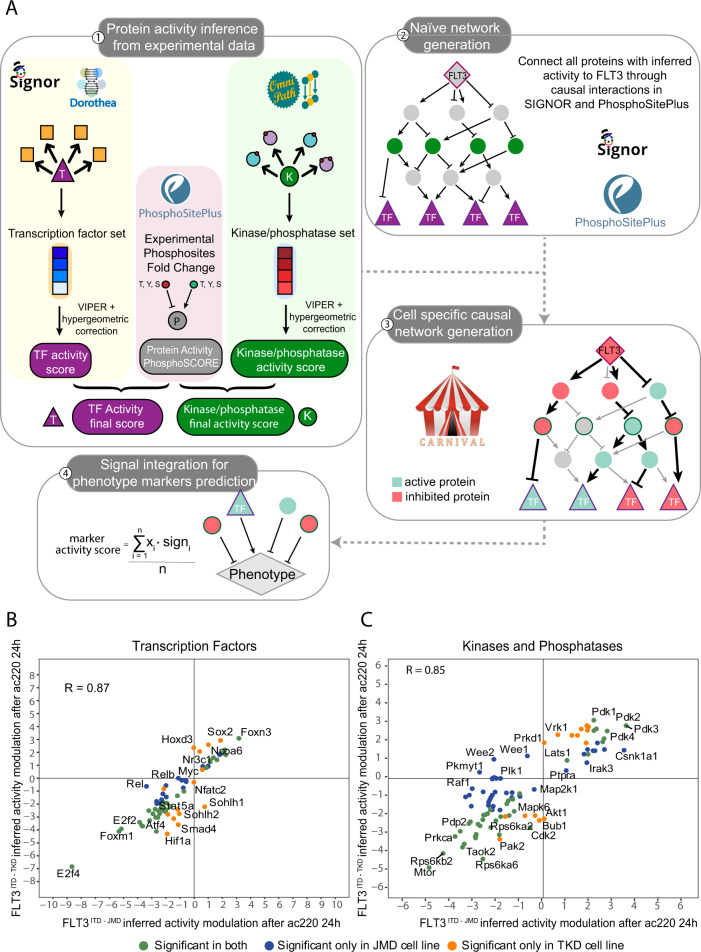
SignalingProfiler strategy predicted an opposite regulation of the WEE1 protein family kinases activity between FLT3^ITD-JMD^ and FLT3^ITD-TKD^ cells. **A** Schematic representation of the *SignalingProfiler* workflow. Step 1. Protein activity of transcription factors, kinases and phosphatases was computed from experimental data using the footprint-based analysis and the “phosphoSCORE” method. When needed, the two scores were averaged. Step 2. Proteins derived from step 1 were linked to FLT3 and to each other’s via paths of causal interactions extracted from PhosphoSitePlus and SIGNOR databases to build a naïve network. Step 3. CARNIVAL was used to search in the naïve network causal circuits coherent with protein activity. More specifically, in the first run we retrieved paths between FLT3 and kinases, phosphatases and substrates, whereas the second run connected all the proteins obtained from the first run with transcription factors. Eventually, the two networks were merged together. Step 4. The activity of protein markers of phenotypes (e.g., apoptosis) were predicted integrating the signal from upstream nodes in each cell-specific optimized network. **B**, **C** Protein activity prediction results. Scatterplots showing the comparison between protein activity predicted from FLT3^ITD-JMD^ (*x*-axis) and FLT3^ITD-TKD^ (*y*-axis) datasets for transcription factors (**B**) and kinases and phosphatases (**C**). Each dot represents a gene/protein, and the color indicates whether the prediction is statistically significant in both cell lines (green) or exclusively in one cell line: ITD-JMD (blue) or ITD-TKD (orange). R indicates Pearson correlation.

Here we implemented “*SignalingProfiler”* (https://github.com/SaccoPerfettoLab/SignalingProfiler/), a generally applicable modeling strategy that takes advantage of previously developed computational approaches [12] to integrate transcriptomics and phosphoproteomics datasets with prior knowledge annotated in public databases such as SIGNOR [13] and PhosphoSitePlus [14] (Fig. 3A and Fig. S5). Briefly:

1. We combined the footprint-based analysis with our newly developed method “Phosphoscore” novel [12] to infer the activity of key proteins: transcription factors (from the transcriptome data) and kinases and phosphatases (from phosphoproteomics data) (Fig. 3A, step 1).
2. We used the causal relations annotated in SIGNOR and PhosphoSitePlus, to build a naïve network connecting (i) FLT3, (ii) inferred kinases and phosphatases, (iii) their substrates and (iv) inferred transcription factors. This network connects proteins through every possible causal path with minimal length, among all possible ones (Fig. 3A, step 2).
3. To retain only causal paths coherent with experimentally-derived proteins’ activities, we exploited CARNIVAL software [15], and we derived FLT3^ITD-JMD^ and FLT3^ITD-TKD^ specific mechanistic models (Fig. 3A, step 3).
4. To in silico validate the results, we inferred the activity of key apoptotic markers as a proxy for the behavior of the two models (Fig. 3A, step 4).

The first step of the *SignalingProfiler* pipeline allowed us to compute the activity of 101 kinases, 22 phosphatases and 70 transcription factors (Fig. 3B, C, Fig. S6 and Table S4). As displayed in Fig. 3C, there is a high correlation between protein activities predicted in the two cell lines (R = 0.85–0.87), with a few exceptions: WEE1, WEE2 and PKMYT1 kinases are predicted to be inactive in the FLT3^ITD-JMD^ and active in the FLT3^ITD-TKD^ cells.

Protein activities of key signaling proteins are then used to feed the CARNIVAL tool together with causal networks (Fig. 3A steps 2 and 3), to obtain two cell-specific models (Figs. S7, S8).

These two graphs are static representations of the remodeling of the signal transduction cascade induced by 24 h of quizartinib treatment. The comparison between sensitive and resistant signaling networks has the potential to reveal potential mechanisms of drug resistance and new therapeutic targets. To this aim, we compared the activity of the nodes inferred by CARNIVAL within the two models. Our analysis revealed that most of the nodes display similar regulation, independently from the ITD insertion site (R = 0.91), whereas few crucial nodes (eg. WEE1, BUB1, RCC1, PLK1) are oppositely regulated or FLT3 ^ITD-TKD^ specific (Fig. S9A).

We next decided to monitor differences of the two FLT3-ITD specific signaling network models at a more granular level (Fig. 3A, step 4). Briefly, we checked whether the two models display differential modulation of a subset of text-mining derived pro-survival and pro-apoptotic proteins (see Methods), in agreement with the phenotypes observed in the two experimental systems. As shown in Supplementary Fig. 9B, FLT3^ITD-TKD^ cells display a stronger activation of pro-survival proteins (especially, MCL1 and BCL2) and inhibition of apoptotic proteins (in particular, BAD and BIM/BCL2L11) compared to FLT3^ITD-JMD^ cells. Interestingly, CDK1 resulted to be the key upstream regulator of four out of five pro-apoptotic and anti-apoptotic proteins (Fig. S9B).

### FLT3-ITD insertion site impacts the WEE1-CDK1 axis and impairs cell cycle progression in TKIs treated cells

**Fig. 4:**
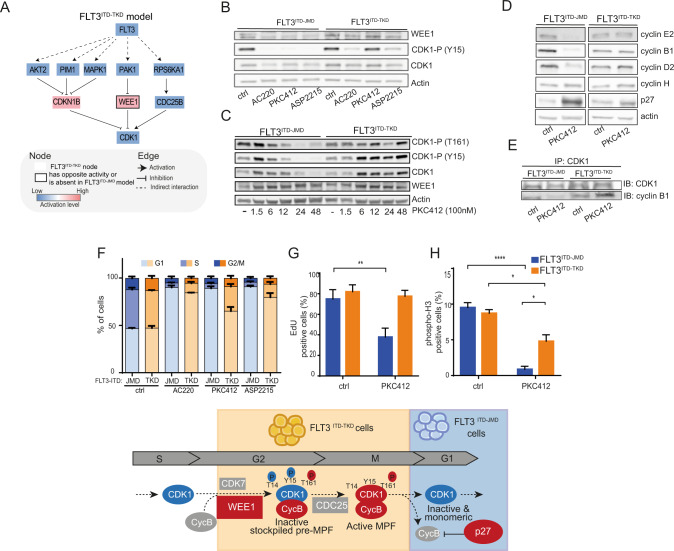
The ITD location affects the WEE1-CDK1 axis and the regulation of the cell cycle upon TKIs treatment. **A** FLT3 - CDK1 signal cascade. FLT3^ITD-TKD^ specific mechanistic model highlighting the regulation of CDK1 downstream of FLT3. Activated proteins are marked in red, whereas inhibited ones in blue. Black bordered nodes display opposite or no regulation in FLT3^ITD-JMD^ model. **B** Representative western blot showing the phosphorylation level of CDK1 on Tyr15 and the protein level of WEE1 kinase in FLT3^ITD-JMD^ and FLT3^ITD-TKD^ BaF3 cells treated for 24 h with 20 nM quizartinib (AC220), 100 nM midostaurin (PKC412) and 50 nM gilteritinib (ASP2215). **C** Representative western blot showing the phosphorylation level of CDK1 on Tyr15, Thr161 and the protein level of WEE1 kinase in FLT3^ITD-JMD^ and FLT3^ITD-TKD^ BaF3 cells treated for the indicated time points with 100 nM midostaurin (PKC412). **D** Representative western blot showing the protein level of different cyclins and p27 inhibitor in FLT3^ITD-JMD^ and FLT3^ITD-TKD^ Ba/F3 cells treated for 24 h with 100 nM midostaurin (PKC412). **E** Representative western blot showing the amount of cyclin B1 isolated by CDK1 immunoprecipitation in FLT3^ITD-JMD^ and FLT3^ITD-TKD^ Ba/F3 cells treated for 24 h with 100 nM midostaurin (PKC412). **F** Cell cycle analysis. Boxplots displaying the percentage of FLT3^ITD-JMD^ (in blue) and FLT3^ITD-TKD^ (in orange) cells in the different phases of the cell cycle as determined by flow cytometry using DAPI labeling, after treatment for 24 h with 20 nM quizartinib (AC220), 100 nM midostaurin (PKC412) and 50 nM gilteritinib (ASP2215). **G** Effect of midostaurin on cell division. FLT3^ITD-JMD^ (blue) and FLT3^ITD-TKD^ (orange) BaF3 cells were treated with 100 nM midostaurin (PKC412) for 24 h. Percentage of cells in division was assessed by EdU labeling and flow cytometry analysis. **H** Bar plot representing the percentage of cells in mitosis. FLT3^ITD-JMD^ (blue) and FLT3^ITD-TKD^ (orange) BaF3 cells were treated with 100 nM PKC412 for 24 h. Cells expressing phospho-H3 (S10) were identified by flow cytometry analysis. WEE1 – CDK1 mechanistic model. **I** Cartoon representing the potential molecular mechanism of chemoresistance suggested by the *SignalingProfiler* analysis. TKI treated FLT3^ITD-JMD^ are stacked in the G1 phase through the accumulation of a monomeric, inactive pool of CDK1, whereas TKI treated FLT3^ITD-TKD^ cells can progress through the cell cycle thanks to the accumulation of the CDK1-Cyclin B complex.

Given the promising role of CDK1 in mediating TKI resistance, we extracted the sub-cascade that leads to its deregulation in both FLT3-ITD models (Fig. 4A, Fig. S10A). As displayed in the diagram in Fig. 4A, CDK1 regulation downstream of FLT3 involves p27/CDKN1B, the kinase WEE1 and the phosphatase CDC25B. Interestingly, the activity of WEE1, a crucial component of the G2-M cell cycle checkpoint [16], is oppositely regulated in the two cell lines (Fig. 3C, Fig. 4A and Fig. S10A) and the inhibitory interaction between WEE1 and CDK1 is FLT3^ITD-TKD^ specific (Figs. S7, S8, S10A). We, therefore, speculate that the WEE1-CDK1 path might play a pivotal role in the FLT3^ITD-TKD^ cells TKI resistance.

Our experiments demonstrated that TKIs treatment differently impacts the abundance of WEE1 in FLT3-ITD cells (Fig. 4B), without affecting its transcript level (Fig. S10B). Consistently, in our phosphoproteomic data, the phosphorylation level of the serine 139, which has been demonstrated to correlate with its degradation [17], is lower in FLT3^ITD-TKD^ cells as compared to FLT3^ITD-JMD^ cells (Fig. S10C and Table S3). In FLT3^ITD-TKD^ cells treated with midostaurin for 24 h, the increased WEE1 protein level determines the hyperphosphorylation of the Tyr15 of CDK1, which is also more phosphorylated on the Thr161 (Fig. 4C). In this triply phosphorylated form, CDK1 is significantly more associated with cyclin B1 (Fig. 4E). Thus, midostaurin treatment in FLT3^ITD-TKD^ cells induces the accumulation of the inactive stockpiled pre-M-phase Promoting Factor (or MPF), the universal mitotic inducer in eukaryotic cells, constituted by the CDK1-Cyclin B1 complex. The formation of this complex is associated with a significant accumulation of proliferating FLT3^ITD-TKD^ cells in the G2-M phase as compared to midostaurin treated FLT3^ITD-JMD^ cells (Fig. 4G–I). By contrast, in FLT3^ITD-JMD^ cells midostaurin treatment for 24 h induces (i) the dephosphorylation of CDK1 at both the tyr15 and thr161 (Fig. 4C); (ii) the accumulation of the p27 inhibitor (Fig. 4D); and the consequent cyclin B1 degradation (Fig. 4D). These data indicate that midostaurin treatment induces a cell state wherein CDK1 is inactive and monomeric (Fig. 4E), and consequently, FLT3^ITD-JMD^ cells accumulate in the G1 phase (Fig. 4F–H).

Taken together, our data indicate that the WEE1-CDK1 axis plays a pivotal role in the TKI sensitivity of FLT3-ITD cells. The FLT3^ITD-TKD^ specific upregulation of WEE1 protects cells against the midostaurin-mediated cell cycle arrest. Indeed, we observed that midostaurin significantly reduces cell proliferation only in FLT3^ITD-JMD^ cells, as revealed by the EdU assay (Fig. 4G). Accordingly, phosphoH3 staining revealed that the percentage of mitotic cells is significantly lower in FLT3^ITD-JMD^ as compared to FLT3^ITD-TKD^ cells upon midostaurin exposure (Fig. 4H). At the molecular level, we observed that cyclin D2, E2, and B1, involved in G1-, S- and G2/M progression, respectively, are significantly down-regulated by midostaurin treatment only in FLT3^ITD-JMD^ cells (Fig. 4D).

Altogether our observations indicate that midostaurin has opposite effects on cell cycle progression in sensitive FLT3^ITD-JMD^ and resistant FLT3^ITD-TKD^ cells (Fig. 4I): TKI treated FLT3^ITD-JMD^ are stacked in the G1 phase through the accumulation of a monomeric, inactive pool of CDK1, whereas TKI treated FLT3^ITD-TKD^ cells can progress through the cell cycle thanks to the accumulation of the CDK1-Cyclin B complex.

### WEE1 kinase inhibition reverts the TKI-therapy resistance of FLT3^ITD-TKD^ cells

Prompted by our observations, we next investigated whether pharmacological inhibition of WEE1 and consequent hyperactivation of CDK1 would potentiate the pro-apoptotic effect of TKIs in FLT3^ITD-TKD^ cells.

**Fig. 5:**
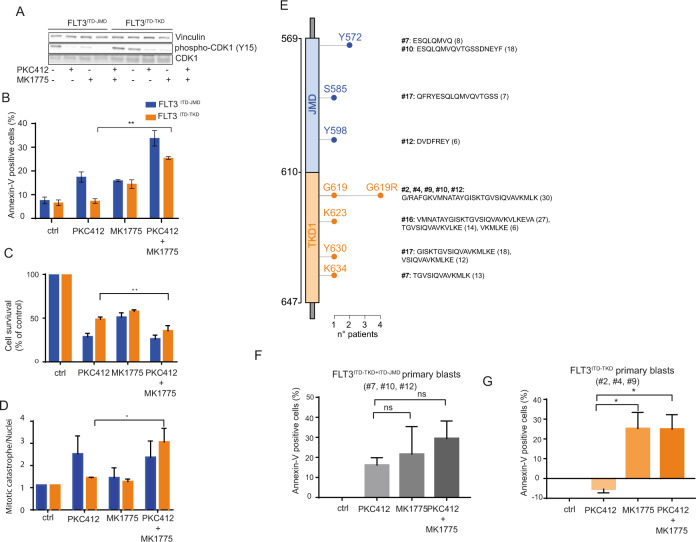
WEE1 kinase inhibition sensitizes FLT3^ITD-TKD^ cells and primary blasts to TKI treatment. **A** Representative western blot showing the effect of the WEE1 inhibitor, adavosertib, on the phosphorylation levels of CDK1 on tyrosine 15. FLT3^ITD-JMD^ and FLT3^ITD-TKD^ BaF3 cells were treated with 100 nM PKC412, 500 nM adavosertib (MK1775) and the combination of both for 24 h. **B** FLT3^ITD-JMD^ (blue) and FLT3^ITD-TKD^ (orange) BaF3 cells were treated with 100 nM midostaurin (PKC412), 500 nM adavosertib (MK1775) and the combination of both for 24 h. Percentage of apoptotic cells was assessed by Annexin-V labeling. **C** FLT3^ITD-JMD^ (blue) and FLT3^ITD-TKD^ (orange) BaF3 cells were treated with 100 nM midostaurin (PKC412), 500 nM adavosertib (MK1775) and the combination of both for 24 h. Cell survival relative to control after treatment was calculated by MTT assay. **D** FLT3^ITD-JMD^ (blue) and FLT3^ITD-TKD^ (orange) BaF3 cells were treated with 100 nM midostaurin (PKC412), 500 nM adavosertib (MK1775) and the combination of both for 24 h. Percentage of mitotic catastrophe was assessed by DAPI labeling of nuclei. **E** Lollipop plot representing the location, amino acid sequence and length of FLT3-ITD mutations in the 9 patients analyzed. 4 patients have ITD located in TKD1 domain (#2, #4, #16, #19), 5 in both TKD1 and JMD domain (#1, #7, #10, #12, #17). Each lollipop length represents the number of patients having an ITD in that position. All mutations were derived from Sanger sequencing from primary patient blasts. **F** Barplot showing the percentage of treatment-induced apoptosis (100 * (dead cells after treatment – death cells in control) / viable cells in control) in patient-derived blasts carrying FLT3-ITD in both the JM and the TK1 domains upon the indicated treatments (patients: #7, #10, #12). Percentage of apoptotic cells was assessed by Annexin-V labeling. **G** Barplot showing the percentage of treatment-induced apoptosis (100 * (dead cells after treatment – death cells in control) / viable cells in control) in patient-derived blasts carrying FLT3-ITD exclusively in the TK1 domain upon the indicated treatments (patients: #2, #4, #19). Percentage of apoptotic cells was assessed by Annexin-V labeling.

Briefly, we treated FLT3^ITD-JMD^ and FLT3^ITD-TKD^ cells with adavosertib (MK1775), a highly selective WEE1 inhibitor [18], separately or in combination with midostaurin. As previously described, adavosertib treatment induces S and/or G2/M cell cycle checkpoints override (Fig. S11A), depending on cancer types [19]. Consistently, we observed that WEE1 inhibition results in a significant accumulation of both FLT3-ITD cells in the S phase (Fig. S11B), wherein CDK1 is dephosphorylated and inactive (Fig. S11C). Apoptotic and cell survival assays showed that WEE1 inhibitor synergizes with midostaurin to trigger cell death of FLT3^ITD-TKD^ cells and to a lesser extent of FLT3^ITD-JMD^ cells (Fig. 5B, C).

Pharmacological inhibition of WEE1 kinase activity and the consequent removal of the G2–M checkpoint through the CDK1 hyperactivation represents an attractive strategy to drive cancer cells to enter into unscheduled mitosis and, arguably, to undergo cell death via alternative mechanisms such as the mitotic catastrophe [20]. In line with this hypothesis, the combined treatment of midostaurin and adavosertib, exclusively, induces the accumulation of cells in the S phase as well as to a lesser extent in the G2-M phase, synergically triggering mitotic cell death in FLT3^ITD-TKD^ cells (Fig. 5D, Fig. S11B).

We next investigated whether FLT3-ITD primary blasts, derived from 9 patients with de novo AML diagnosis, could benefit from the combined treatment of midostaurin and WEE1 inhibitor. First, blasts were isolated from peripheral blood of ITD-positive AML patients (Fig. S12). As expected, the molecular landscape of FLT3-ITD mutation is heterogeneous (Fig. 5E). We excluded one patient carrying an atypical insertion sequence in the JMD domain. Then, we classified our cohort of FLT3-ITD patients in two main groups according to the ITD insertion site (Fig. 5E and Fig. S13). This approach enabled us to obtain two subgroups: 4 FLT3^ITD-TKD^ patients (carrying the ITD in the TKD domain), 4 FLT3^ITD-JMD+ITD-TKD^ patients (carrying the ITD in both the JMD and TKD domain). We considered only patients with a single insertion, reaching three patient per subgroup. Unexpectedly, in our cohort, no patient carrying the ITD only in the JMD domain was found. Remarkably, genetic stratification based on the ITD localization reflected the drug-response phenotype (Fig. S13): midostaurin alone significantly triggers apoptosis only in FLT3^ITD-JMD+ITD-TKD^ blasts (Fig. 5F), while no beneficial effects were observed in FLT3^ITD-TKD^ blasts (Fig. 5G). This result suggests that the pro-apoptotic effect of the ITD insertion within the JM domain is dominant over the ITD-TKD counterpart. This observation is consistent with the retrospective analyses of the CALGB 10603/RATIFY trial showing that patients with insertions in JMDsole and/or JMD/TKD1 had a significantly improved overall survival and a lower cumulative incidence of relapse compared to patients with insertion sites in TKD1sole [6].

Remarkably, both the pharmacological inhibition of WEE1 alone and the combined treatment with midostaurin trigger apoptosis of FLT3^ITD-TKD^ positive blasts, restoring their sensitivity to TKI therapy (Fig. 5G).

Our results provide novel evidence that the WEE1-CDK1 axis represents a promising therapeutic target to revert drug resistance in patients carrying the ITD mutation in the TKD of FLT3 that currently cannot benefit from midostaurin treatment.

## Discussion

The insertion site of the ITD mutations significantly impacts the ability of FLT3 inhibitors, including midostaurin, to trigger cell death in both cell lines and primary blasts [7, 9, 21–23]. Consistently with previous observations, here we show that the beneficial effect of TKIs is restricted to FLT3-ITDs located in the juxtamembrane domain (JMD), but not to FLT3-ITD in the TKD region. Indeed, ITDs in the TKD alone predispose to chemoresistance and relapse, necessitating a clearer understanding of the mechanisms underlying TKI sensitivity toward the development of more effective and targeted treatments. Although the upregulation of the anti-apoptotic myeloid cell leukemia 1 protein (MCL-1) has been proposed to be involved in TKI resistance in cells carrying the insertion in the TKD domain [9], novel, complementary, genotype-specific therapeutic approaches are still missing.

We have reported here the first unbiased, large-scale, multi-layered analysis aimed at describing the molecular mechanisms underlying the different sensitivity to TKI therapy of cells carrying FLT3-ITD mutations in the TKD or JMD domains. The main objective of this study is the identification of new potential therapeutic targets increasing the efficacy of TKI therapy in FLT3^ITD-TKD^ patients.

Our quantitative transcriptome, proteome and phosphoproteome analysis provide an integrated picture of TKIs-dependent molecular events. We speculate that a complex rewiring of signaling pathways may be the cause of the different sensitivity of FLT3-ITD cells to TKIs treatments. To address this point, we implemented a computational pipeline dubbed *SignalingProfiler*, that integrates readouts of transcriptome and phosphoproteome studies, with prior evidence annotated in public repositories to produce cell-specific networks representing the remodeling of signal transduction cascade at the PTM-resolution level induced by quizartinib. The observed inhibition of canonical pathways immediately downstream of FLT3 [24] as well as the presence of well-characterized gene products whose mutation are involved in AML progression or relapse (e.g., NPM1, CEBPA, KRAS, PTPN11) [25], confirm the clinical relevance of our models.

Although based on previously developed tools such as CARNIVAL [15] and VIPER [26], SignalingProfiler incorporates novel features such as the PhosphoSCORE calculation method, enabling for a comprehensive integration of the phosphoproteomic data; and the simulation of apoptosis biomarkers, which provide the *in-silico* validation of the results.

This approach revealed a novel mechanism of resistance relying on differential regulation of the WEE1-CDK1 axis. Here we demonstrate that midostaurin has opposite effects on cell cycle progression in FLT3-ITD cells. TKI treated FLT3^ITD-JMD^ are blocked in the G1 phase through the accumulation of a monomeric, inactive pool of CDK1, resulting more sensitive to apoptosis induction. On the other side, TKI treated FLT3^ITD-TKD^ cells can progress through the cell cycle thanks to the WEE1-dependent upregulation of the CDK1-Cyclin B complex, resulting less prone to undergo to apoptosis. Consistently, increased levels of WEE1 have been shown to correlate with tumor progression and poor progression-free survival [27]. Here, we demonstrated that deregulation of the WEE1-CDK1 axis represents a crucial mechanism of resistance to TKI therapy in FLT3-ITD positive cells and patient-derived primary blasts. Remarkably, the ability of FLT3^ITD-TKD^ cells to undergo apoptosis in response to TKI therapy was completely rescued by pharmacological inhibition of WEE1. This observation provides support for WEE1 inhibitors to be used in combination therapies with TKIs to improve the clinical outcomes of FLT3^ITD-TKD^ patients.

The molecular mechanisms through which the different FLT3-ITD insertion sites may affect the activity of the WEE1-CDK1 axis in response to TKI treatment is still under investigation. Our hypothesis is that the different ITDs location likely impacts the structure of the receptor tyrosine kinase FLT3, influencing its ability to recruit different signaling proteins (data not shown). This in turn may impinge on the WEE1-CDK1 axis, ultimately affecting the TKI-mediated cell cycle arrest and apoptosis induction. Further investigations will be required to confirm this hypothesis.

Interestingly, although we analyzed a small cohort of FLT3-ITD patient-derived primary blasts, our analysis confirms that insertions in the TKD sole have a significantly worse clinical outcomes compared to blasts with insertion sites in both the JM and the TK domains [6].

In conclusion, the results of this work highlight limitations in the current practice toward the treatment of FLT3-ITD positive patients affected by AML and open-up opportunities for additional, more effective and patient-specific therapeutic strategies. Here, we speculate that these pre-clinical results create the basis of new trials that might change the clinical reality for AML patients. We suggest that FLT3-ITD patients, at diagnosis, should be stratified according to the ITD insertion site into prognostically relevant FLT3-ITD subgroups. Midostaurin maintenance therapy should be critically evaluated in case of FLT3-ITD located within the TKD1 domain and synergistic combination therapies should be used to rationally manipulate the WEE1-CDK1 axis, triggering cell death, through mechanisms that are yet to be defined.

Finally, our work demonstrates how unbiased, system-level studies have the potential to accelerate the discovery of more granular, patient-specific mechanisms of disease and chemoresistance toward the identification of more effective therapeutic approaches.

## Supplementary information

Supplementary material

Figure S1

Figure S2

Figure S3

Figure S4

Figure S5

Figure S6

Figure S7

Figure S8

Figure S9

Figure S10

Figure S11

Figure S12

Figure S13

Dataset 1

Dataset 2

Dataset 3

Dataset 4

Dataset 5

## Data Availability

The mass spectrometry proteomics data have been deposited to the ProteomeXchange Consortium via the PRIDE (34723319) partner repository with the dataset identifier PXD033953.
