# Supplementary material for: A key role of the WEE1-CDK1 axis in mediating TKI-therapy resistance in FLT3-ITD positive acute myeloid leukemia patients
Source: Leukemia. 2022 Dec 12;37(2):288–97. doi: 10.1038/s41375-022-01785-w (PMC9898030; doi:10.1038/s41375-022-01785-w)
Supplement: Supplementary file 1 — Supplementary material [file 41375_2022_1785_MOESM1_ESM.docx]

**Supplementary material**

**A key role of the WEE1-CDK1 axis in mediating TKI-therapy resistance in FLT3-ITD positive acute myeloid leukemia patients**

Giorgia Massacci^1*^, Veronica Venafra^1*^, Sara Latini^1^, Valeria Bica^1^, Giusj Monia Pugliese^1^, Felix Klingelhuber^2^, Natalie Krahmer^2^, Thomas Fischer^3,4^, Dimitrios Mougiakakos^5^, Martin Boettcher^4,5^, Livia Perfetto^1,6,#^ and Francesca Sacco^1,7,#^

**Supplementary Materials and Methods**

**Cell culture**

Mouse Ba/F3 cells expressing ITD-JMD and ITD-TKD constructs were provided by courtesy of T. Fischer. The cells were cultured in RPMI 1640 medium (Hyclone, Thermo Scientific, Waltham, MA) supplemented with 10% heat-inactivated fetal bovine serum (ECS0090D Euroclone, Italy, MI ), 100 U/ml penicillin and 100 mg/ml streptomycin (Gibco 15140122), 1 mM sodium pyruvate (Sigma-Aldrich, St. Louis, Missouri, United States, S8636) and 10 mM 4-(2-hydroxyethyl)-1-piperazineethanesulfonic acid (HEPES) (Sigma H0887). These cells were chosen as experimental system according to the following criteria: (i) they faithfully recapitulate the essential biological features of FLT3-ITD induced transformation in AML as factor-independent growth, colony formation, resistance to apoptosis, and oncogenic signaling ^13,14^; (ii) homogeneous and experimentally easy to manipulate system; (iii) commonly used by the leukemia scientific community as model systems to characterize the oncogenic potential, signaling and sensitivity to tyrosine kinase inhibitors ^7,15–20^; (iv) enable to investigate the effect of the different FLT3-ITD insertion sites isolated from real AML patients in the same genetic background (FLT3^ITD-TKD^ and FLT3^ITD-JMD^ cells are isogenic) in-vitro and in-vivo ^7^ and (v) reflect the differential TKI sensitivity of patient derived blasts carrying the different FLT3-ITD mutations ^7,17,21^. Quizartinib (Selleck chemicals, S1526), midostaurin (Selleck chemical, S8064), gilteriitnib (Selleck chemicals, S7754), MK1775 (MedChemExpress, HY-10993) were used at 5-50 nM, 100 nM, 50 nM and 500 nM respectively.

**Immunoblot analysis**

BaF3 cells were seeded at a concentration of 500.000 cells/ml and treated as indicated. After treatments cells were centrifuged and washed in PBS 1x. Next, cells were lysed in ice-cold lysis buffer (150 mM NaCl, 50 mM Tris–HCl, pH 7.5, 1% Nonidet P-40, 1 mM EGTA, 5 mM MgCl_2_, and 0.1% SDS) supplemented with 1 mM PMSF, 1 mM ortovanadate, 1 mM NaF, protease inhibitor mixture 1×, inhibitor phosphatase mixture II 1×, and inhibitor phosphatase mixture III 1× and incubated for 30 min. Protein lysates were separated at 13,000g for 30 min. The total protein concentration was determined using the Bradford reagent (Biorad, 5000006). Protein extracts were denatured and heated at 95°C for 10 min in NuPAGE LDS Sample Buffer (Thermo Fisher Scientfic, NP0007) and a buffer contained DTT as a reducing agent (NuPAGE Sample Reducing Agent) (Thermo Fisher Scientfic, NP0004). Proteins were resolved using 4–15% Bio-Rad Mini-PROTEAN TGX/CRITERION polyacrylamide gels (Bio-Rad 4561084). Proteins were transferred to Trans-Blot Turbo Mini Nitrocellulose Membranes using a Trans-Blot Turbo Transfer System (Bio-Rad, 17001918), and the nonspecific binding membranes were saturated in blocking solution (5% skimmed milk powder, 0.1% Tween 20 in 1× TBS) at room temperature for 1 hour. Saturated membranes were incubated overnight with primary antibodies diluted in BSA 5% (anti-phospho FLT3 1:1000, CST 3464S; anti-phosphoSTAT5 1:1000, Abcam ab32364; anti-phosphoERK1/2 1:1000, CST 9101; anti-FLT3 1:1000, CST 3462; anti-STAT5 1:1000, Abcam ab16276; anti-ERK1/2 1:1000, CST 4695; anti-phospho CDK1 (Y15), CST 9111S; anti-CDK1/2 1:1000, Santa Cruz sc. 53219; anti-Wee1 1:1000, Abcam ab273016; anti-actin 1:3000, Sigma A2066). HRP-conjugated secondary antibodies (Goat Anti-Mouse IgG (H+L)-HRP Conjugate 1:3000, BIORAD 1721011) were diluted in blocking solution and used for the detection of the primary antibodies. Chemiluminescence was detected using Clarity Western ECL Blotting Substrates (Bio-Rad) and the Las-3000 Imaging System (Fujifilm). Band densities were quantified using ImageJ and normalized to the loading control.

**MTT assay**

Cell viability was measured using the Cell Proliferation Kit I (MTT) (Roche). Cells were treated as indicated for 20 hours. Then, MTT was added to the cells and incubated for 4 hours at 37 ◦C. Solubilization Solution was used to dissolve the formazan crystals during an overnight incubation. Finally, the plates were read at 590nm using a microplate reader (Bio-Rad).

**Sample preparation for proteomic and phosphoproteomic analysis**

Cell lysis was performed by adding SDC lysis buffer containing 4% (w/v) SDC, 100 mM Tris -HCl (pH 8.5). Samples were immediately boiled at 95° for 5 minutes and sonicated in bioruptor for 10 cycles at high intensity 30s on/30s off. Next, protein concentration was quantified by BCA assay. For the proteome preparation, we used the inStageTip (iST) method for the proteome preparation ^45^. Briefly, 50µg of protein for each sample was diluted in 2% SDC buffer and 1% trifluoroacetic acid (TFA). SDBRPS tips were washed with i) 100 µl acetonitrile (ACN), ii) 100 µl of 30% methanol and 1% TFA and iii) 150 µl of 0.2 % TFA centrifuging tips at 1000 xg for 3 minutes. Samples were loaded onto equilibrated columns and spin at 1000 xg for 10 minutes. SDBRPS tips were washed with i) 100 µl of 1% TFA in ethyl acetate, ii) 100 µl of 1% TFA in isopropanol and iii) 0.2% TFA. For the elution of proteins, we used a buffer containing 80% ACN, 5% NH_4_OH in MilliQ water. Samples were centrifuged at 1000 xg for 4 minutes and concentrated by SpeedVac at 45° for ~45 minutes. Finally, samples were dissolved in 10μl of a buffer containing 2% ACN and 0.1% TFA. Phosphoproteome preparation was performed by the EasyPhos workflow as previously described ^46^. Briefly, equal protein amount (>750 μg) was diluted in 750 μl of ACN and 250 μl of EP enrichment buffer containing: 36% TFA and 3mM KH_2_PO_4_. Samples were mixed at 2000 xg for 30 s to clear precipitates, centrifuged at 20.000 xg for 15 minutes and transferred to a 2 ml deep-well plate. To enrich phosphosites, TiO_2_ beads were used. 12:1 (beads: protein) for each samples were weighted out and resuspended in EP loading buffer containing 80% ACN and 6% (v/v) TFA. Activated TiO_2_ beads were added to each sample and incubated for 5 minutes at 40° C at 2000 rpm. Next, beads were pelleted at 2000 xg for 1 minute and supernatants (non-phosphosites) were discarded. Beads were resuspended in 500μl of EP wash biffer consisting of 60% ACN, and 1% TFA twice and transferred to clean tube/plate. Four additional washed with EP wash buffer were performed mixing at 2000 rpm for 3 seconds. After wash, beads were resuspended in 75 μl of EP transfer buffer (80% ACN, 0.5% Acetic acid), transferred on top of C8 stage tips (double layer) and spin to dryness at 1000 xg for 5 minutes. Phosphopeptides were eluted with 30 μl of EP elution buffer containing 200 μl of NH_4_OH and 800 μl of 40% ACN into PCR tubes. Immediately, samples were concentrated in SpeddVac at 45° C for 20 minutes. During SpeedVac, SDBRPS tips (triple layer) were equilibrated using i) 100 μl ACN, ii) 100 μl 30% methanol and 1% TFA and iii) 150 μl 0.2% TFA. After SpeedVac, SDBRPS loading buffer (1% TFA in isopropanol) was added to the samples. Next, phosphopetides were loaded onto equilibrated SDBRPS StageTips and washed with i) 100 μl 1% TFA in EtOAc, ii) 100 μl of 1% TFA in isopropanol and iii) 150 μl of 0.2% TFA. After wash phosphopetides were eluted into clean PCR tubes with a buffer containing 60% ACN and 5% NH_4_OH. After SpeedVac at 45° C for 30 minutes, phosphopetides were resuspended in 10μl of a buffer containing 2% ACN and 0.1% TFA.

**Mass spectrometry analyses**

The peptides and the phosphopeptides were desalted on StageTips and separated on a reverse phase column (50 cm, packed in-house with 1.9-mm C18- Reprosil-AQ Pur reversed-phase beads) (Dr Maisch GmbH) over 120 min or 140 min (single-run proteome and phosphoproteome analysis respectively). After elution, peptides were electrosprayed and analyzed by tandem mass spectrometry on a Orbitrap Exploris 480 (Thermo Fischer Scientific). The instrument was set to alternate between a full scan followed by multiple HCD based fragmentations scans for a total cycle time of up to 1 s.

**RNAseq analysis**

BaF3 cells were cultured in growth medium at the density of 500.000 cell/ml. Next, AC220 was added at a concentration of 20nM and incubated for 24 hours. Then, cells were centrifuged at a speed of 300 x g, washed in PBS and total RNA was isolated from the harvested cells using RNeasy micro kit (Qiagen, Hilden, Germany 74004) and was quantified using the Qubit 2.0 fluorimetric Assay (Thermo Fisher Scientific). Libraries were prepared from 100 ng of total RNA using the QuantSeq 3' mRNA-Seq Library Prep Kit FWD for Illumina (Lexogen GmbH, Vienna, Austria) and their qualities were assessed by using screen tape High sentisivity DNA D1000 (Agilent Technologies, Santa Clara, California, United States). Libraries were sequenced on a NovaSeq 6 000 sequencing system using an S1, 100 cycles flow cell (Illumina Inc., San Diego, California, United States). Illumina novaSeq base call (BCL) files were converted into fastq file by bcl2fastq (version v2.20.0.422). Sequence reads were trimmed using bbduk software (bbmap suite 37.31) in order to remove adapter sequences, poly-A tails and low-quality end bases (regions with average quality below 6). Alignment was performed with STAR 2.6.0a ^47^ on mm10 reference assembly obtained from cellRanger website (Ensembl assembly release 93). The expression levels of genes were determined with htseq-count 0.9.15 (Ref. 29) by using mm10 Ensembl assembly (release 93) downloaded from cellRanger website.

**Proteome and Phosphoproteome Data processing**

Raw mass spectrometry data were analyzed in the MaxQuant environment ^48^, version 1.5.1.6, employing the Andromeda engine for database search. Proteome and phosphoproteome samples were analysed together by specifying two separate groups and setting group specific parameters for each sample type. MS/MS spectra were matched against the *Mus musculus* UniProtKB FASTA database (September 2014), with an FDR of < 1% at the level of proteins, peptides and modifications. Enzyme specificity was set to trypsin, allowing for cleavage N-terminal to proline and between aspartic acid and proline. The search included cysteine carbamidomethylation as a fixed modification. Variable modifications were set to N-terminal protein acetylation and oxidation of methionine as well as phosphorylation of serine, threonine tyrosine residue (STY) for the phosphoprotemic samples. MaxQuants Label free Quantification method and a minimum ration count of two was used for the total proteome samples. For proteome and phosphoproteome analysis, where possible, the identity of peptides present but not sequenced in a given run was obtained by transferring identifications across liquid chromatography (LC)-MS runs (‘match between runs’). For phosphopeptide identification, an Andromeda minimum score and minimum delta score threshold of 40 and 17 were used, respectively. Peptides had to be fully tryptic in both proteome or phosphoproteme samples and up to two or four missed cleavages were allowed for protease digestion, respectively.

**Proteome and Phosphoproteome Bioinformatics Data Analysis**

Bioinformatic analysis was performed in the Perseus software environment ^49^. Statistical analysis of proteome and phosphoproteome were performed on logarithmized intensities for those values that were found to be quantified in any experimental condition. Phosphopeptides intensities were normalized by subtracting the median intensity of each sample. Student t-Test with a permutation-based FDR cutoff of 0.07 and S0 = 0.1 was performed to identify significantly modulated proteins and phosphopetides between two different conditions. Categorical annotation was added in Perseus in the form of GO biological process (GOBP), molecular function (GOMF), and cellular component (GOCC), KEGG pathways and kinase substrate motifs (extracted from HPRD). Concerning the kinase substrate motifs, we performed a 1D annotation enrichment analyses to identify statistically significant enriched kinase-substrates motifs in AC220 treated cells ^26^. Multiple hypothesis testing was controlled by using a Benjamini-Hochberg FDR threshold of 0.05.

**EdU incorporation assay**

For the proliferation assay cells were seeded at the concentration of 500.000 cells/ml and treated for 24 hours with 100nM PKC412. During the last two hours of incubation 5-ethylnyl-2′-deoxyuridine (EdU) was added at a concentration of 10 μM. After incubation, 1x10^6^ cells were centrifuged at 300g for 5 min and then washed in PBS1X. The Click-iT® reaction to detect EdU positive cells was performed according to the manufacturer’s instructions. Percentage of cell in division was assessed by flow cytometry.

**Phospho-H3 labelling**

Cells were seeded at a concentration of 500.00 cells/ml and treated with 100nM PKC412 for 24 hours. After treatment, 1x10^6^ cells were centrifuged at 300g for 5 min and then washed once in PBS1X. Cells were fixed with 70% ethanol overnight at 4°C. Next, cells were centrifuged at 300xg for 10 min and washed once with PBS1X+2%BSA. 500µl of PBS1X+1% saponin was added and incubated for 15 min at room temperature to permeabilize cells. After a wash with PBS1X+2%BSA, cells were incubated with the anti-phospho-H3 antibody (Abcam AB267372) diluted 1:500 in PBS1X+1%BSA+0.5% saponin for 90 min at room temperature. After a wash in PBS1X+1%BSA, cells were incubated with 100ul of an anti-rabbit Alexa Fluor 455 diluted 1:200 in PBS1X+1%BSA+0.5%saponin for 1h at room temperature. After the incubation, cells were washed with PBS1X twice. The percentage of phosphor-H3 positive cells were quantified by flow cytometry.

**Primary patient blast analyses**

Peripheral blood (PB) samples from AML patients were obtained upon patient’s informed consent and in accordance with the declaration of Helsinki (ethics committee approval number: 115/08). The integration site of the FLT3-ITD mutation was determined as previously described ^17^. Briefly, RNA was prepared from PBMCs using the RNeasy Mini Kit (Qiagen, Germany), reverse-transcribed to cDNA using the SuperScript reverse transcriptase system (ThermoFisher Scientific), and the FLT3-ITD region was amplified by PCR (fw-primer: GCAATTTAGGTATGAAAGCCAGC, rev-primer: CTTTCAGCATTTTGACGGCAACC). PCR products were re-purified using the QIAquick PCR purification kit (Qiagen) and subjected to Sanger sequencing (using the same fw-primer) by Eurofins (Luxembourg).

To classify patients according to the ITD localization, we first translated the raw FASTA files obtained from Sanger sequencing using all the possible reading frames using R package “Biostrings” ^50^. Using blastp ^51^, we then aligned all the translated sequences to the JMD and TKD domain sequences of canonical FLT3, as annotated in UniProtKB. Manual evaluation of the alignment allowed us to identify the insertion site and duplicated sequences, as displayed in **Figure 5E**.

Mononuclear cells from the PB were obtained using Ficoll-Paque (GE Healthcare, Chicago, IL). Cryoconserved PBMCs from 12 patients were cultured at a density of 5x10^5^ /mL in RPMI-1640 (Sigma-Aldrich, St. Louis, MO) supplemented with 10% FCS (c.c.pro, Germany), 2 mM L-glutamine (Sigma-Aldrich), and 40 U/mL Penicillin-Streptomycin (ThermoFisher Scientific) for 24h in absence or presence of PKC412, MK1775 or a combination of both.

Viability of the AML blasts was determined by flow cytometry using Annexin V – APC and 7AAD together with the Annexin V staining buffer according to the manufacturers’ instruction (Biolegend, San Diego, CA). Prior to viability staining, samples were stained with fluorochrome-coupled antibodies after blocking with human IgG (Gamunex, Grifols, Barcelona, Spain). Samples were recorded on a Cytek NL-3000 spectral flow cytometer. First, blasts were gated based on the CD45/SSC distribution and on FSC-A/FSC-H parameters to identify single cells population. Then, blasts were gated according to the expression of typical blast markers (CD33, CD34, CD13, CD117). Data was analyzed using FlowJo V10 (Becton-Dickinson, Franklin Lakes, NJ).

**Statistics**

All the experiments have been conducted in at least 3 independent replicates obtained from 3 cell line batches (n = 3). Data are presented as means ± standard error of the mean (SEM). Multiple comparisons between three or more groups were performed using one-way or two-way ANOVA. Statistical significance between two groups was estimated using the unpaired t test assuming a two-tailed distribution. Statistical significance is defined as *p < 0.05; **p < 0.01; ***p < 0.001. All statistical analyses were performed using Prism 7 (GraphPad).

**Code availability**

The SignalingProfiler code is available as R package at <https://github.com/SaccoPerfettoLab/SignalingProfiler/>.

**Causal interaction database download**

We downloaded all the causal interactions available for *Mus musculus* (TaxID = 10090) and *Homo sapiens* (TaxID = 9606) from the SIGNOR ^25^ and PhosphoSitePlus® ^52^ resources. SIGNOR 2.0 datasets were downloaded via rest API and refer to December 2021. Interactions in SIGNOR annotated with ‘down-regulates’, ‘up-regulates’ or ‘unknown’ were assigned values -1, 1 and 0, respectively.

Causal phosphorylations were extracted from PhosphoSitePlus® ^28^ by manually downloading and combining two independent tables: (i) kinase-phosphosite interactions (‘Kinase_Substrate_Dataset.gz’), (ii) regulatory role of phosphosites on protein (‘Regulatory_sites.gz’). Tables were joined using, as key, the UNIPROT ID and the modified residue. We, next, manually mapped the content of the ‘ON_FUNCTION’ column (representing regulatory role of phosphosites) into 1, -1, 0 values.

The so manipulated datasets were, then, combined together and filtered to retain interactions with a defined regulatory effect (-1 or 1).

The results of this pipeline are two causal interactomes accounting for 19,310 and 25,948 interactions in *Mus musculus* and *Homo sapiens*, respectively.

**Protein activity prediction**

*Footprint-based analysis*

Transcription Factors-target genes collection was retrieved from DoRothEA R Package (v. 1.6.0, organism *Mus musculus,* confidence: A) ^53^ and from SIGNOR (filtering for transcriptional regulations). Kinase-substrates and phosphatase-substrate collections were retrieved from Omnipath ^54^. To estimate kinases and phosphatases’ activity from substrates and transcription factors’ activity from target genes, we used the VIPER algorithm ^43^. We used as phosphosite and gene level statistic (VIPER parameters) their experimental fold change. We set the eset.filter parameter to FALSE. We included proteins with at least 1 measured transcript (or phosphosite). We retained only proteins with enrichment *p-value* < 0.05 in at least one cell line. We obtained the inferred activity of 51 transcription factors, 94 kinases and 20 phosphatases.

Hypergeometric test implemented in RVenn package (v. 1.1.0) was used to derive the p-value that a protein can be significantly enriched by chance. For each protein, we used enrichment_test function with measured genes in each regulon as *set1*, significant genes in each regulon as *set2* and all measured genes as *univ*. Log10(pvalue) scaled in [0,1] range was used to weight each protein VIPER score in order to give less importance to proteins with fewer significantly modulated targets in experimental data.

*PhosphoSCORE*

To maximize the use of phosphoprotemic data, we implemented a novel approach to infer the activity of phosphoproteins being target of (de)phosphorylation modifications (PhosphoSCORE) by combining information about the regulatory role of different phosphosites and their experimental fold-change.

We derived the Quizartinib-induced activity modulation of proteins that are target of (de)phosphorylation from the relative phosphoproteomics data and from the regulatory role of phosphosites parsed from SIGNOR and PhosphoSitePlus (as described in the Causal interaction database section), using the formula:

$$substrate activity=\frac{1}{n}\sum_{i=1}^{n} sign_{i}*\mathrm{FC}_{i}$$

where: *n* is the number of phosphosites regulating a target, *sign_i_* is the regulatory role of phosphosite (1 or -1) and *FC*_i_ is the experimental fold change of phosphosites significantly modulated in at least one cell line.

The regulatory role of phosphosites was inferred from the mouse and the human datasets and combined by orthology mapping, using the blastp software ^51^.

This process allowed us to predict the activity of 7 additional kinases, 19 transcription factors, 2 phosphatases and 70 proteins with other molecular functions. For 3 transcription factors and 11 kinases both VIPER weighted score and PhosphoSCORE were present, in this case we averaged the two scores.

The complete list of inferred protein activities is provided in **Supplementary Table S4**.

**Cell – specific naïve causal network generation**

Murine causal interactions from SIGNOR and PhosphoSitePlus were converted in a graph using ‘igraph’ R package (v. 1.2.10). We, then, extracted a subnetwork containing: (i) all the shortest paths from FLT3 to kinases, phosphatases and substrates; (ii) kinase – substrate and phosphatase-substrates interactions; (iii) all the shortest paths from kinases, phosphatases and substrates to transcription factors. We, thus, obtained a naïve network containing 871 nodes and 3422 edges. In this step, we choose a path connecting two proteins considering only its length and not its causal meaning: as such, there may be two paths with same minimal length, but one it is activatory and one inhibitory.

**CARNIVAL**

For each cell line, we optimized the naïve network on inferred protein activity values using CARNIVAL R package ^30^. CARNIVAL filters the naïve network retaining causal paths coherent with the activity of start and end nodes, , resulting in just one path among every pair of proteins. The naïve network was pre-processed to: (i) remove incoming edges in FLT3 and (ii) remove feedback loops. We, then, performed two runs of CARNIVAL: The first run starting from the FLT3 to kinases, phosphatases and substrates; the second from all nodes present in the output of the previous run to transcription factors. FLT3 activity was assigned a -1 value, since quizartinib (AC220) inhibits its activity, whereas the activity of the remaining nodes was assigned as described in the *Protein activity prediction* section*.*

The networks obtained from the two runs were merged to generate two final, cell-specific networks linking FLT3 to transcription factors, namely the FLT3^ITD-JMD^ model (210 nodes and 363 edges) and the FLT3^ITD-TKD^ model (201 nodes and 322 edges). The two networks are publicly available for browsing at:

The two networks are publicly available for browsing at:

- FLT3^ITD-JMD^ model: <https://www.ndexbio.org/viewer/networks/41d05e72-d6b2-11ec-b397-0ac135e8bacf>

-FLT3^ITD-TKD^ model: <https://www.ndexbio.org/viewer/networks/75d9e6f5-d6b2-11ec-b397-0ac135e8bacf>

**Text mining approach**

We used a text-mining approach to query the literature database, Europe PMC, to identify research articles characterizing pro-apoptotic and pro-survival proteins associated to FLT3-ITD AML. The query is: ("pro-apoptotic" OR "anti-apoptotic" OR "pro-survival" OR "anti-survival") AND (TITLE:"FLT3-ITD" AND (TITLE:"AML" OR TITLE:"Acute Myeloid Leukemia"))AND (OPEN_ACCESS:y) AND (PUB_TYPE:"Research-article" OR PUB_TYPE:"report"). We derived a list of eleven pro- or anti-apoptotic proteins (Table S5).

**Phenotype marker prediction**

To *in silico* validate the results, we extracted from SIGNOR and PhosphoSitePlus direct (one step) connections between nodes in the optimized models and phenotype markers derived from the text mining approach. We were able to connect only five out of eleven proteins, since (i) some proteins were already in the network (e.g. MYC); (ii) some biomarkers didn’t have any direct link (e.g. PIM1, XIAP, BCL2L10); (iii) some proteins displayed contradictory literature evidence (e.g. PARP1, BIRC5).

We integrated the signal on each marker to derive its activity modulation after quizartinib. The impact of each regulator over the apoptotic marker was computed multiplying its activation state, as inferred from the experimental data (as described in the *Protein activity prediction* section), by the sign of regulation, namely -1 for inhibitions and +1 for activations. Finally, all the effects on each marker were averaged to derive the activity score.

**Figure S1. High coverage and reproducibility of proteome and phosphoproteome data.** (**A-C**) Number of quantified transcripts (**A**), proteins (**B**) and phosphosites (**C**) in biological replicates of the indicated experimental conditions. (**D-F**) Heatmap showing the Pearson correlation coefficients between the different biological replicates in the trascriptome (**D**), proteome (**E**) and phosphoproteome (**F**) datasets.

**Figure S2. Transcriptome, proteome and phosphoproteome comparative analysis. A-B)** Correlation analysis between protein and mRNA levels in FLT3^ITD-JMD^ cells (**A**) and FLT3^ITD-TKD^ cells (**B**). Proteins (dots) significantly modulated both at proteome and trascriptome level are marked in violet, whereas those modulated exclusively at the proteome level are indicated in blue. The pie chart details corresponding percentages. **C-D)** Correlation analysis between protein and phosphorylation levels in FLT3^ITD-JMD^ cells (**C**) and FLT3^ITD-TKD^ cells (**D**). Phosphosites (dots) modulated by quizartinib (AC220) treatment and belonging to proteins modulated in quantity (proteome level) are represented in purple, whereas those modulated only at the phosphorylation level are marked in green.

**Figure S3. Comparative analysis of quizartinib-induced changes at the transcriptome, proteome and phosphoproteome levels in FLT3^ITD-JMD^ and FLT3^ITD-TKD^ cells.**

**(A-C)** Donut charts indicating the percentage of quizartinib significantly modulated transcripts (**A**), proteins (**B**) and phosphosites (**C**) in FLT3^ITD-JMD^ and/or FLT3^ITD-TKD^ cells. For the analytes modulated in FLT3^ITD-JMD^ and FLT3^ITD-TKD^, the comparison of transcript, protein and phosphosite level is shown in a scatterplot with Pearson Correlation.

**(E-G)** Comparison of mRNA (**E**), protein (**F**) and phosphosites (**G**) fold change between quizartinib (AC220)-treated FLT3^ITD-JMD^ (x-axis) and FLT3^ITD-TKD^ (y-axis) cell lines. Each dot corresponds to a transcript, protein or phosphosite quantified in both cell lines. Analytes can be significantly modulated by quizartinib (AC220) in both cell lines (blue), or exclusively in one cell line: FLT3^ITD-JMD^ (light blue) or FLT3^ITD-TKD^ (orange). Global Pearson correlation is shown.

(**G-H-I**) Unsupervised hierarchical clustering (Pearson correlation distance) of the log2 intensity of more than 11,000 transcripts (**G**), 5,000 proteins (**H**) and 16,000 phosphosites (**I**), as indicated.

**Figure S4. Global pathways modulation in quizartinib treated FLT3^ITD^ cells.** Two-dimensional annotation enrichment analysis. Pathways modulated in quizartinib treated FLT3^ITD-TKD^ cells (y-axis) at the transcriptome (**A**), proteome (**B**), phosphoproteome (**C-D**) level in comparison with quizartinib treated FLT3^ITD-JMD^ cells (x-axis) (Benjamin Hochberg FDR < 0.05). Each dot represents a specific KEGG pathway or GO-Biological Process (GO-BP) term. Groups of related pathways or GO-BP are labeled with the same color, as described in the inset. Position scores of the pathways at the transcriptome and proteome level are indicated in the x and y axes, respectively ^1^. Negative values indicate downregulation, whereas positive values upregulation. (**E**) Schematic representation of the fatty acids oxidation; for each enzyme the corresponding quizartinib-induced change in mRNA and protein concentration in FLT3^ITD-JMD^ and FLT3^ITD-TKD^ cells is shown. (**F**) Kinase overrepresented in significantly modulated phosphosites identified in FLT3^ITD-JMD^ (blue) and FLT3^ITD-TKD^ (orange) cells.

**Figure S5. Mechanistic cell – specific causal model construction workflow.**

Detailed description of the *Signaling Profiler* pipeline described in Figure 4. **Step 1.** Protein activity of transcription factors (purple triangle), kinases and phosphatases (green circles) was inferred from experimental data combining the footprint-based analysis and the ‘phosphoSCORE' technique. In the first case, protein activity is derived from the modulation of its downstream targets using the VIPER statistical tool ^2^ transcription factors’ activity is calculated from transcriptomic data whereas kinases and phosphatases’ activity from phosphoproteomic data. VIPER output is corrected through a hypergeometric test. In the second case, our de novo developed PhosphoSCORE technique exploits the modulated phosphosites of a protein and their regulatory role (as extracted from SIGNOR ^3^ and PhosphoSitePlus repositories^4^ to compute its activity (PhosphoSCORE). When a protein is assigned to both scores, they are averaged to obtain a final protein activity score.

**Step 2.** Assembly of a network of causal interactions linking FLT3 and proteins characterized in step 1, to build a naïve network. Briefly, we exploited causal interactions annotated in the SIGNOR and PhosphoSitePlus resources to extract all the shortest paths from FLT3 receptor (purple diamond) to kinases and phosphatases (dark green-bordered diamonds). Similarly, we retrieved direct relationships between kinases/phosphatases and their substrates (light green-bordered hexagon). Finally, we searched for the shortest paths from kinases, phosphatases and substrates to transcription factors (purple-bordered triangle). In step 2 we considered exclusively directionality and distance and not the regulatory role of each interaction (up/down regulation).

**Step 3.** CARNIVAL ^5^ was used to search in the naïve network circuits coherent with protein activity inferred in Step 1 (bold black edges): mint green nodes are predicted up-regulated after quizartinib (AC220) treatment, whereas red nodes are down-regulated. Because quizartinib (AC220) causes FLT3 inhibition, the receptor activity was set to -1 (repressed). We executed two runs of CARNIVAL: run 1 retrieved coherent paths linking FLT3 to kinases, phosphatases and substrates, run 2 linking proteins derived from run 1 with transcription factors. The two networks were, eventually, joint to obtain, for each cell line, a final mechanistic model recapitulating the signal cascade downstream of FLT3, rewired by quizartinib (AC220). **Step 4.** To functionally interpret the two cell – specific causal networks, we searched in SIGNOR and PhosphoSitePlus the direct connections between final network nodes and markers of phenotypes of interest (e.g., BCL2, BAD, etc. were considered markers of apoptosis). Finally, we computed the activity of each marker averaging all the activation state (x_i_) of input nodes multiplied for their regulation sign (sign_i_).

**Figure S6. Protein activity prediction from experimental data in quizartinib treated FLT3^ITD^ cells.** (**A-B**) Volcano plots show the modulation of all STAT5A target genes that were used, by the VIPER tool ^6^, to infer its activity, after quizartinib (AC220) treatment in FLT3^ITD-JMD^ (**A**) and FLT3^ITD-TKD^ cells (**B**). X-axis represents log2 fold change of regulated transcripts multiplied by the sign of regulation (-1 for inhibition, 1 for activation of transcription). Y-axis represents the significance of the log fold change (-log10 pvalue). In each grey panel is shown the regulation of significant transcripts by STAT5A.

**(C)** Scatterplot shows the comparison between protein activity predicted from FLT3^ITD-JMD^ (x-axis) and FLT3^ITD-TKD^ (y-axis) datasets for proteins with molecular function different from TF, kinase or phosphatase. Each dot represents a protein, and the color indicates whether the prediction is statistically significant in both cell lines (green) or exclusively in one cell line: FLT3^ITD-JMD^ (blue) or FLT3^ITD-TKD^ (orange). R indicates Pearson correlation.

**Figure S7. FLT3^ITD-JMD^ specific causal­­ network.** Causal network representing the quizartinib-induced signal rewiring in FLT3^ITD-JMD^ cell line. Color of nodes represents activated (red) or inhibited (blue) proteins after the treatment. Shape of nodes reflects molecular function: parallelograms are phosphatases, rectangles are transcription factors, circles are kinases and hexagons are other phosphorylated proteins. Target arrow shape represents activatory (arrow) or inhibitory (T shape) interactions. Black edges represent (de)phosphorylations occurring at phosphosites measured in the experimental data. Additional details are further described in the inset.

**Figure S8. FLT3^ITD-TKD^ specific causal­­ network.** Causal network representing the quizartinib-induced signal rewiring in FLT3^ITD-TKD^ cell line. Color of nodes represents activated (red) or inhibited (blue) proteins after the treatment. Shape of nodes reflects molecular function: parallelograms are phosphatases, rectangles are transcription factors, circles are kinases and hexagons are other phosphorylated proteins. Target arrow shape represents activatory (arrow) or inhibitory (T shape) interactions. Black edges represent (de)phosphorylations occurring at phosphosites measured in the experimental data. Additional details are further described in the inset.

**Fig. S9. (A) Comparison of CARNIVAL inferred activities in FLT3 ITD models.** Scatterplot shows the comparison between CARNIVAL inferred protein activity from FLT3^ITD-JMD^ (x-axis) and FLT3^ITD-TKD^ (y-axis) models. Each dot represents a node or a group of nodes (with same combination of activity values), the dot color indicates the presence in both cell models (green) or exclusively in one cell model: FLT3^ITD-JMD^ (blue) or FLT3^ITD-TKD^ (orange) and the dot size reflects the number of nodes in each group. R indicates Pearson correlation. **(B) Activity inference of apoptosis markers and activation state of their regulators**. Barplot showing the activity score predicted for pro-survival proteins (purple) and pro-apoptotic proteins (green) in FLT3^ITD-JMD^ (blue) and FLT3^ITD-TKD^ (orange) cell lines. In each grey panel, heatmaps show the activation state of upstream regulators of each apoptosis marker. Positive and negative regulators are displayed in green and red, respectively. For each regulator, the barplot on the right reflects the absolute value of the difference in activity between FLT3^ITD-JMD^ and FLT3^ITD-TKD^ models.

**Figure S10**. (**A**) FLT3 - CDK1 signal cascade. FLT3^ITD-JMD^ specific mechanistic model highlighting the regulation of CDK1 downstream of FLT3. Activated proteins are marked in red, whereas inhibited ones in blue. Black bordered nodes display opposite or no regulation in FLT3^ITD-TKD^ model. (**B**) Bar plot showing the Log2 expression level of Wee1 mRNA quantified in the transcriptome analysis in FLT3^ITD-JMD^ (blue) and FLT3^ITD-TKD^ (orange) cells after quizartinib (AC220) treatment. (**C**) Bar plot showing the Log2 intensity values of the Wee1 phosphorylation on Serine 139 quantified in the phosphoproteomic analysis after 1.5 hour and 24 hours of quizartinib (AC220) treatment in FLT3^ITD-JMD^ (blue) and FLT3^ITD-TKD^ (orange) cells.

**Figure S11.** (**A**) Schematic representation of the WEE1-CDK1 axis functional role in cell cycle progression. (**B**) Cell cycle analysis. Boxplots displaying the percentage of FLT3^ITD-JMD^ and FLT3^ITD-TKD^ cells in the different phases of the cell cycle as determined by flow cytometry using DAPI labeling, after treatment for 24 hours with 100nM midostaurin (PKC412) and/or 500nM adavosertib (MK1775). (**C**) Representative western blot showing the phosphorylation level of CDK1 on Tyr15, Thr161 and the protein level of CDK1 in FLT3^ITD-JMD^ and FLT3^ITD-TKD^ Ba/F3 cells treated for 24 hours with 100nM midostaurin (PKC412) and/or 500nM adavosertib (MK1775).

**Figure S12.** Gating strategy used for the viability assay analysis of primary blasts samples. Blasts were gated based on the CD45/SSC distribution (“blast gate”), for single cells (by FSC-A/FSC-H) and for typical blast markers heterogeneously expressed between patients (CD33, CD34, CD13, CD117). Gates were set according to an unstained/CD45-single stained control. Data was analyzed using FlowJo V10 (Becton-Dickinson, Franklin Lakes, NJ).

**Figure S13. (A)** Barplots showing blasts treatment specific cell death (*100 * (dead cells after treatment – death cells in control) / viable cells in control*) in each FLT3^ITD-TKD^ patient after 100 nM midostaurin (PRC412), 500 nM Wee1 inhibitor (MK1775) and combination of both. **(B)** Barplots showing blasts treatment specific cell death (*100 * (dead cells after treatment – death cells in control) / viable cells in control*) in each FLT3 ^ITD-JMD+ITD-TKD^ patient after 100 nM midostaurin (PRC412), 500 nM Wee1 inhibitor (MK1775) and combination of both.

**References**

1. Cox, J. & Mann, M. 1D and 2D annotation enrichment: a statistical method integrating quantitative proteomics with complementary high-throughput data. *BMC Bioinformatics* **13 Suppl 16**, 1–11 (2012).

2. Alvarez, M. J. *et al.* Network-based inference of protein activity helps functionalize the genetic landscape of cancer. **48**, 838–847 (2016).

3. Licata, L. *et al.* SIGNOR 2.0, the SIGnaling Network Open Resource 2.0: 2019 update. *Nucleic Acids Res.* **48**, D504–D510 (2020).

4. Hornbeck, P. V. *et al.* PhosphoSitePlus, 2014: Mutations, PTMs and recalibrations. *Nucleic Acids Res.* **43**, D512–D520 (2015).

5. Liu, A. *et al.* From expression footprints to causal pathways: contextualizing large signaling networks with CARNIVAL. *npj Syst. Biol. Appl.* **5**, 1–10 (2019).

6. Alvarez, M. J. *et al.* Functional characterization of somatic mutations in cancer using network-based inference of protein activity. *Nat. Genet.* **48**, 838–847 (2016).
