# Supplementary figures and images for: A key role of the WEE1-CDK1 axis in mediating TKI-therapy resistance in FLT3-ITD positive acute myeloid leukemia patients
Source: Leukemia. 2022 Dec 12;37(2):288–97. doi: 10.1038/s41375-022-01785-w (PMC9898030; doi:10.1038/s41375-022-01785-w)

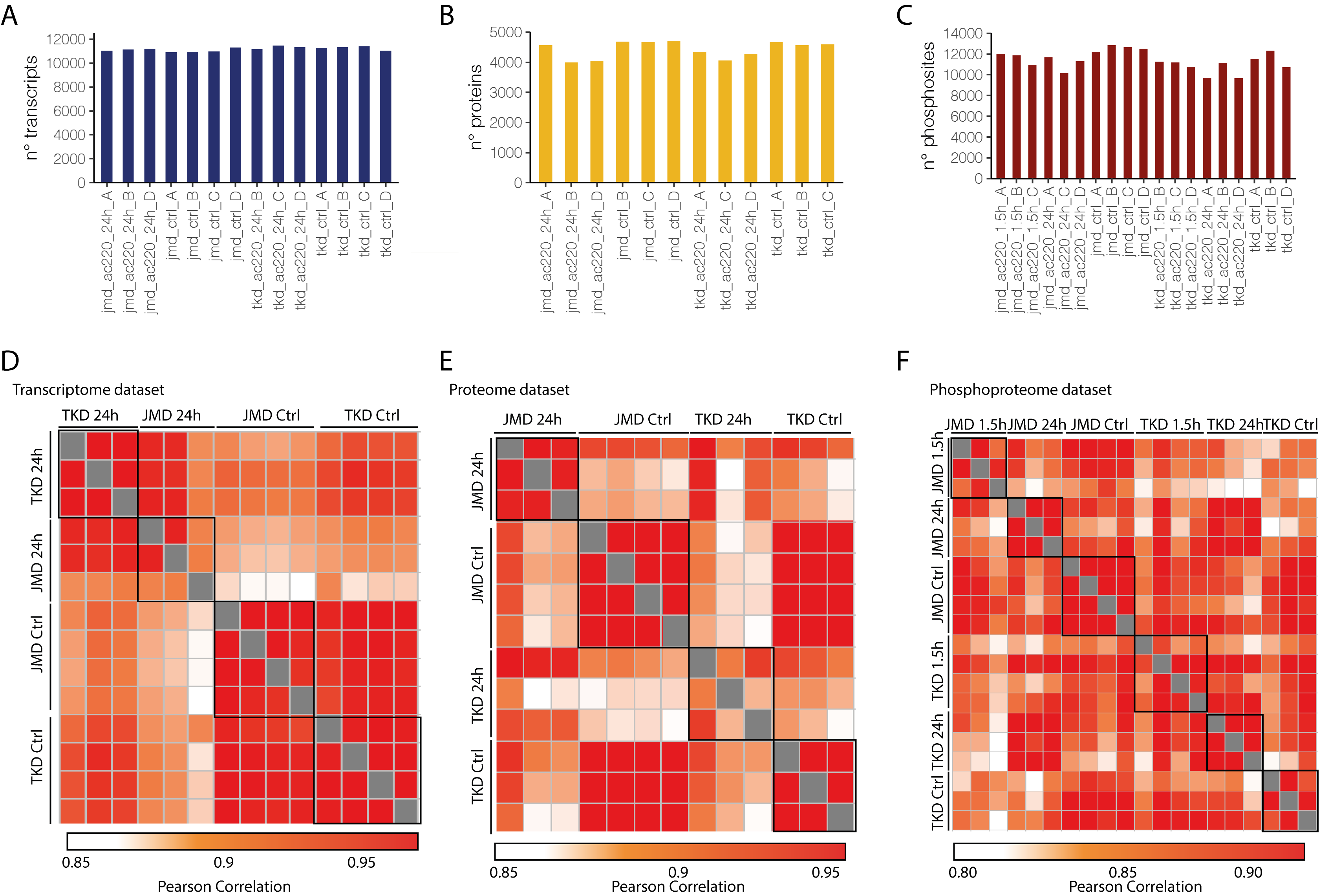

Supplement: Supplementary file 2 — Figure S1 [file 41375_2022_1785_MOESM2_ESM.tif]

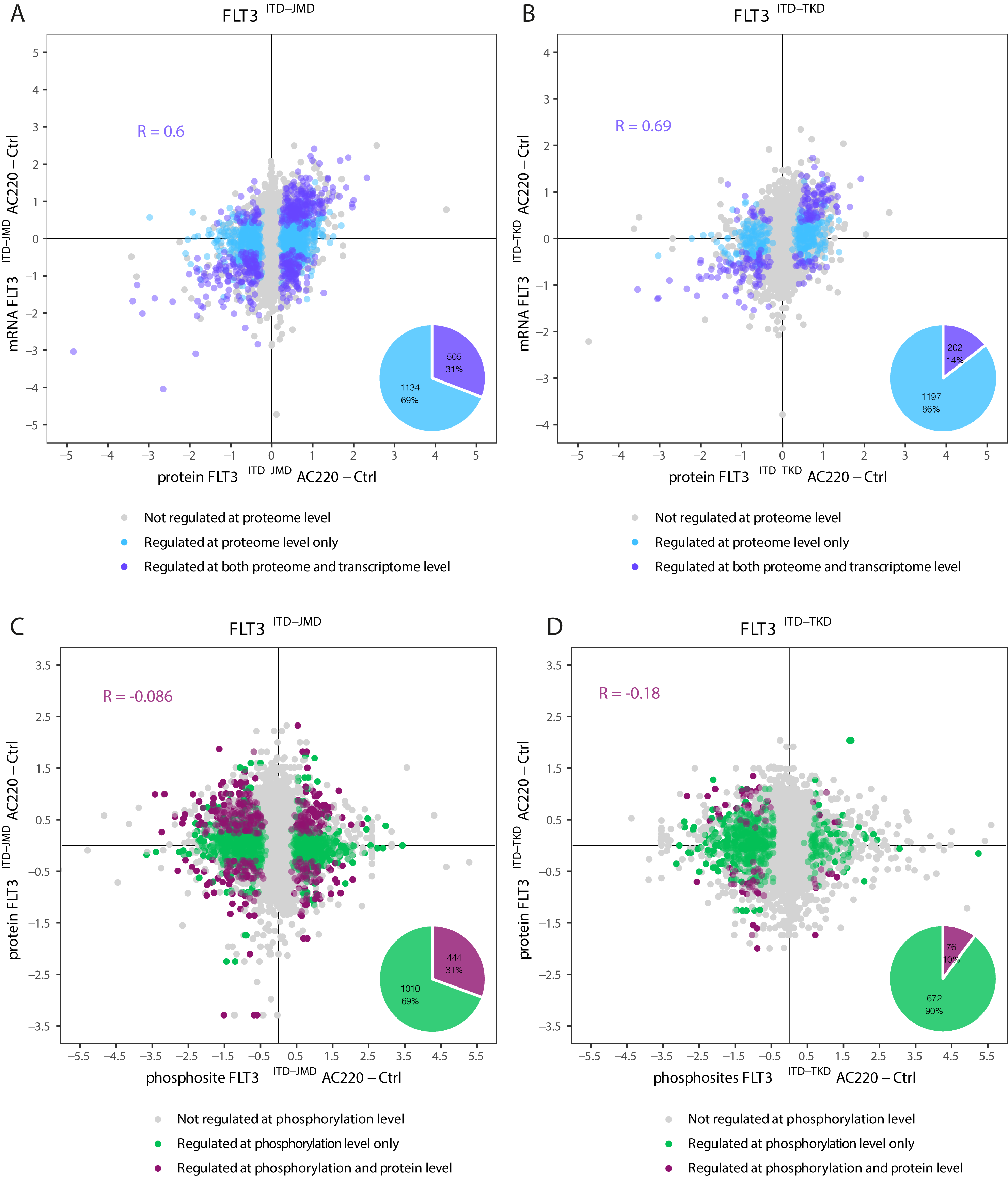

Supplement: Supplementary file 3 — Figure S2 [file 41375_2022_1785_MOESM3_ESM.tif]

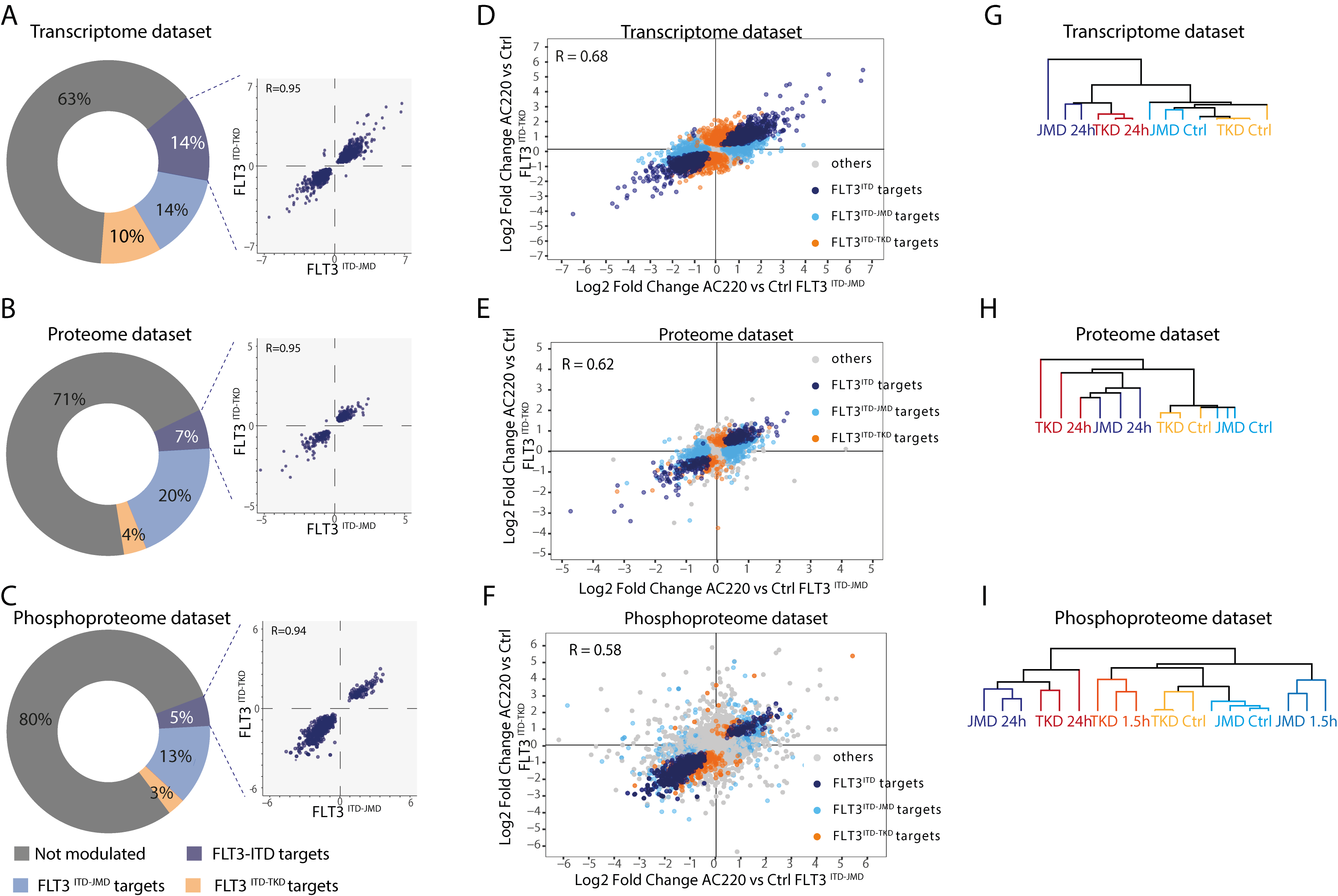

Supplement: Supplementary file 4 — Figure S3 [file 41375_2022_1785_MOESM4_ESM.tif]

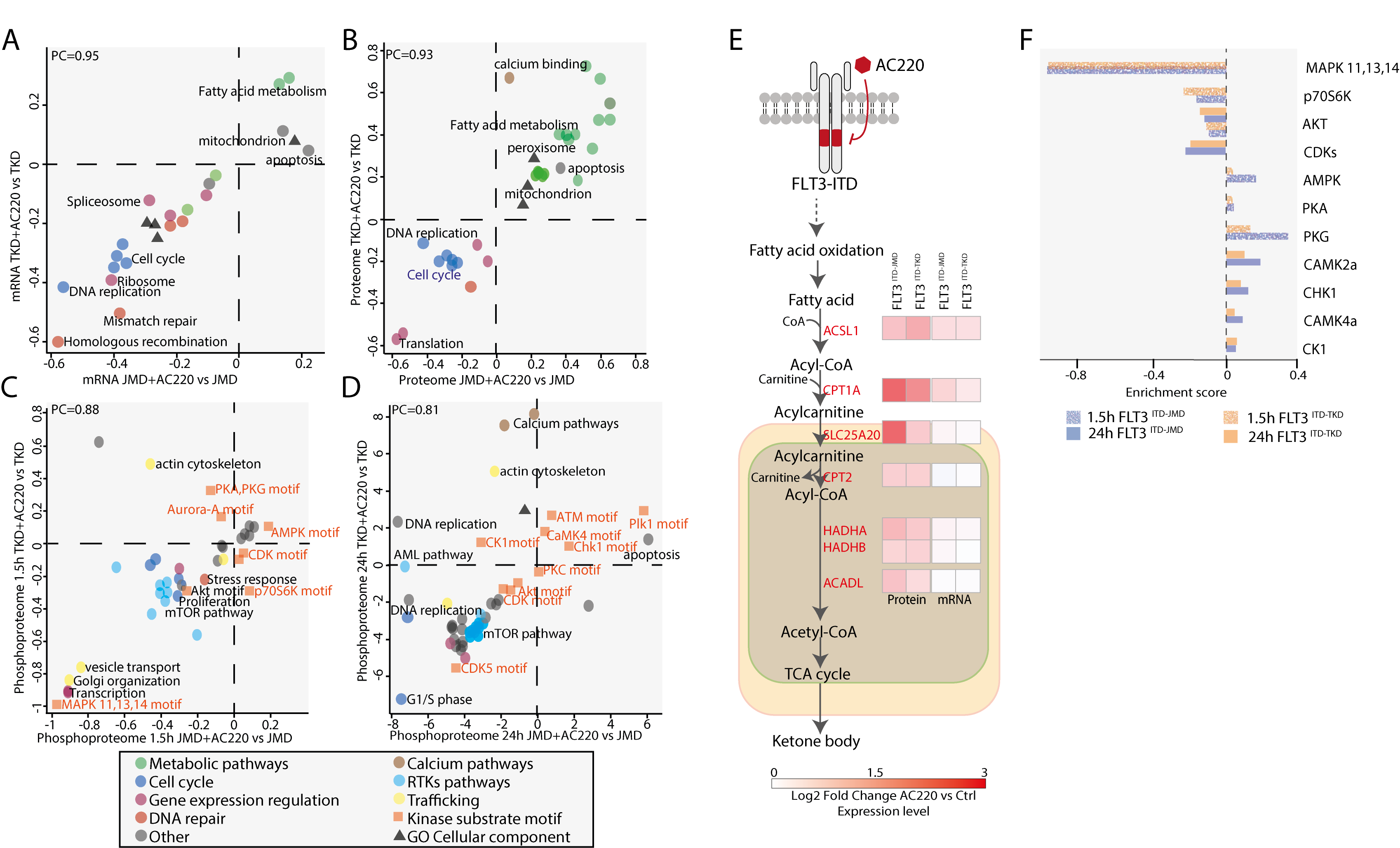

Supplement: Supplementary file 5 — Figure S4 [file 41375_2022_1785_MOESM5_ESM.tif]

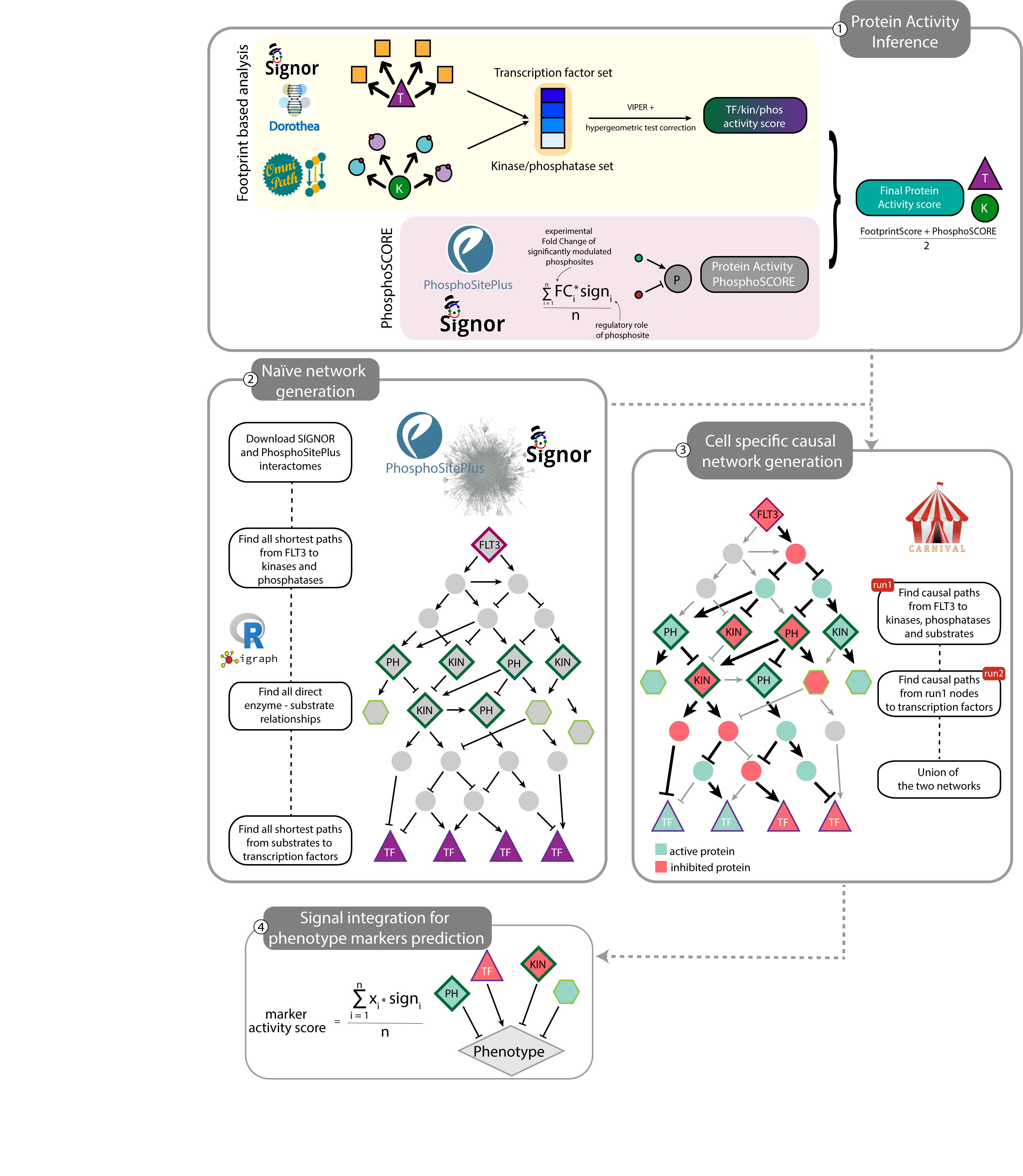

Supplement: Supplementary file 6 — Figure S5 [file 41375_2022_1785_MOESM6_ESM.tif]

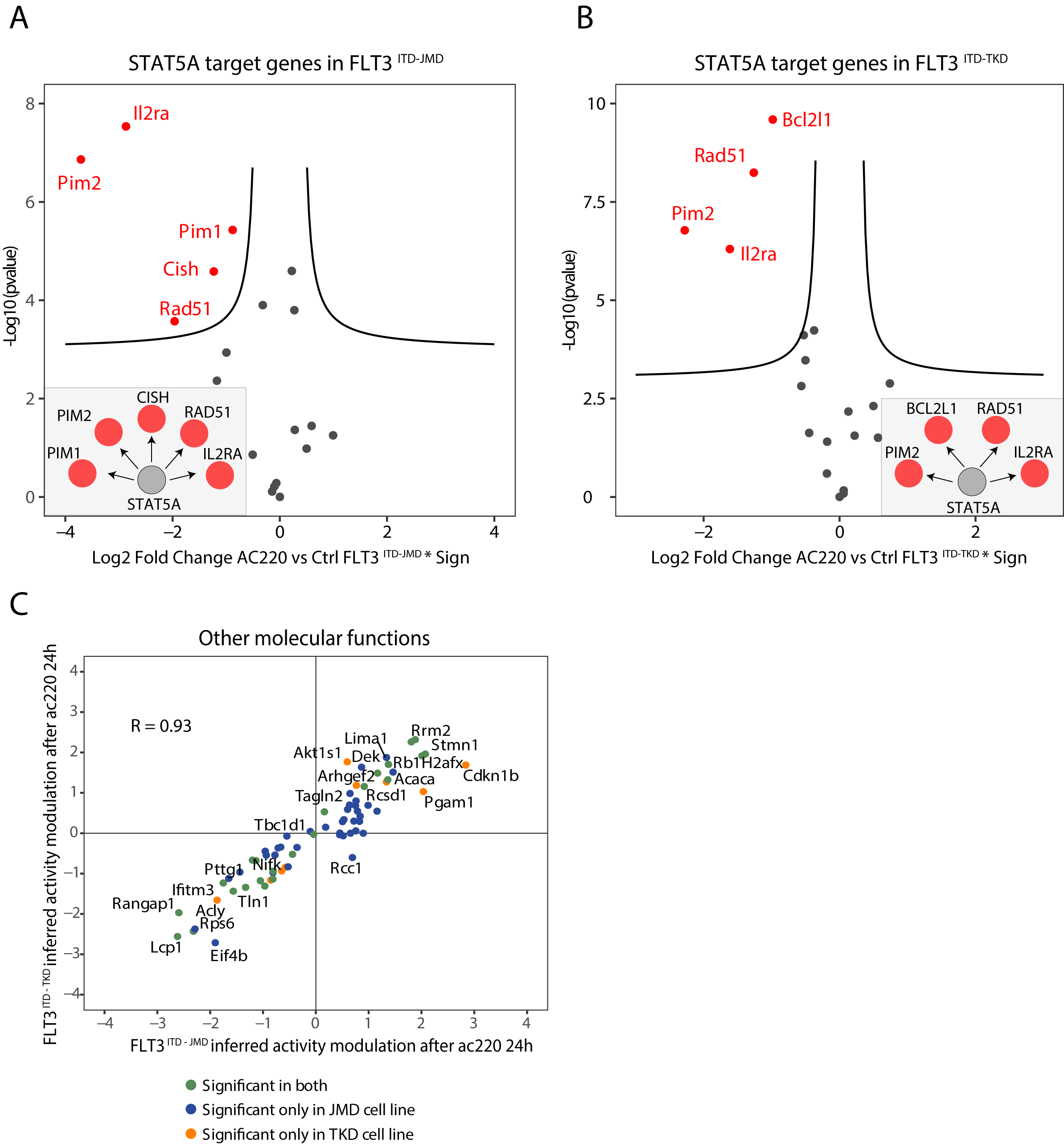

Supplement: Supplementary file 7 — Figure S6 [file 41375_2022_1785_MOESM7_ESM.tif]

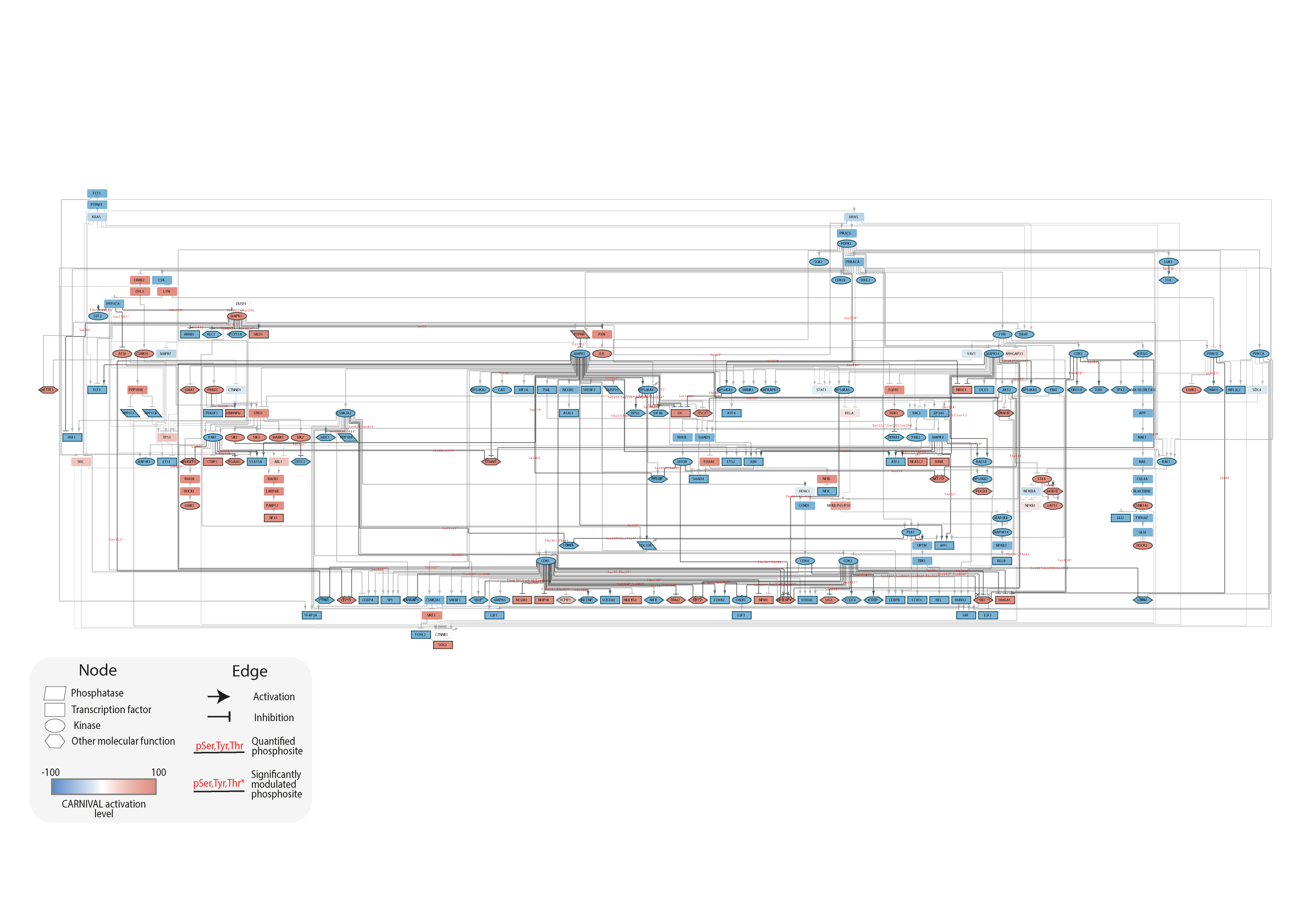

Supplement: Supplementary file 8 — Figure S7 [file 41375_2022_1785_MOESM8_ESM.tif]

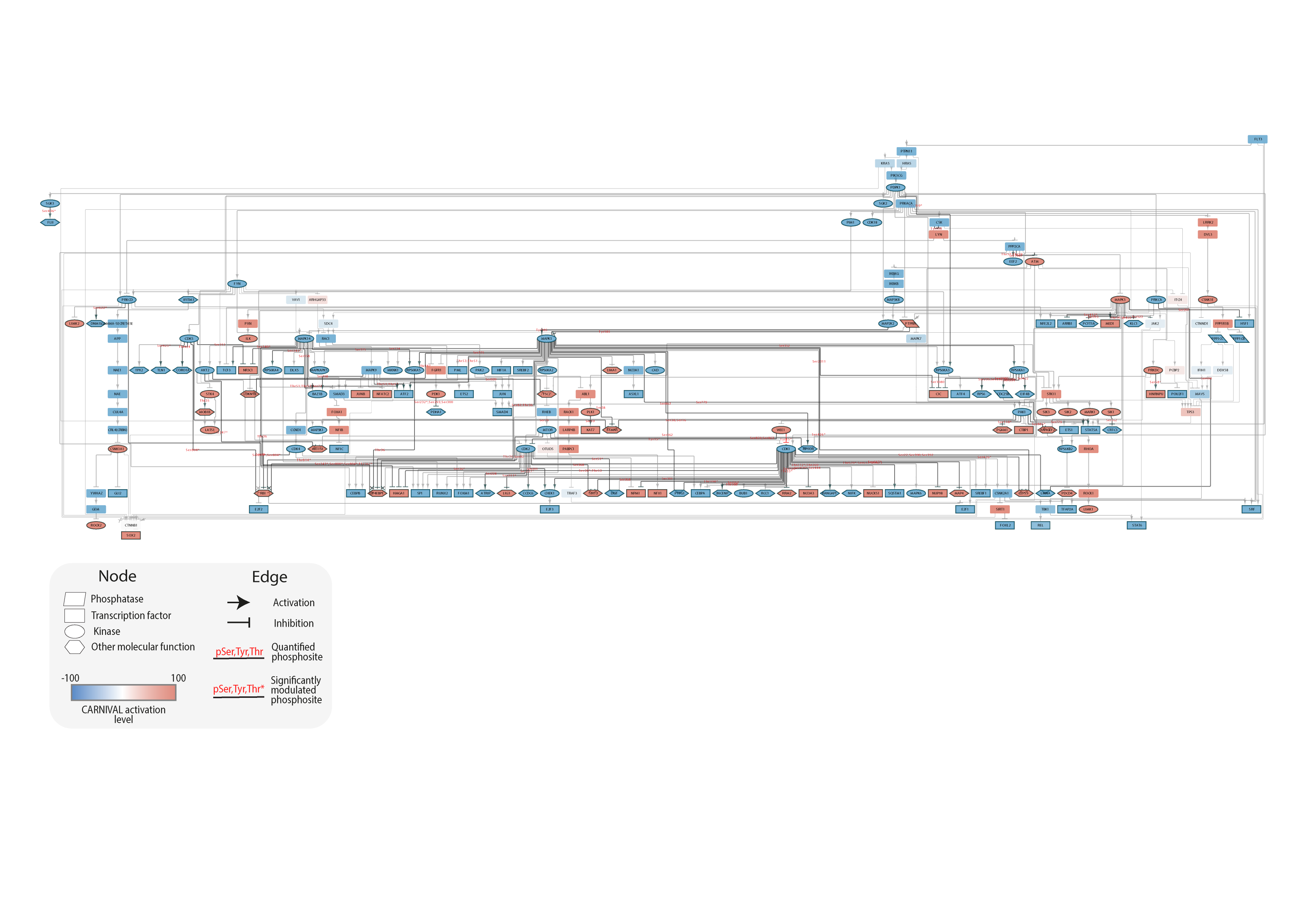

Supplement: Supplementary file 9 — Figure S8 [file 41375_2022_1785_MOESM9_ESM.tif]

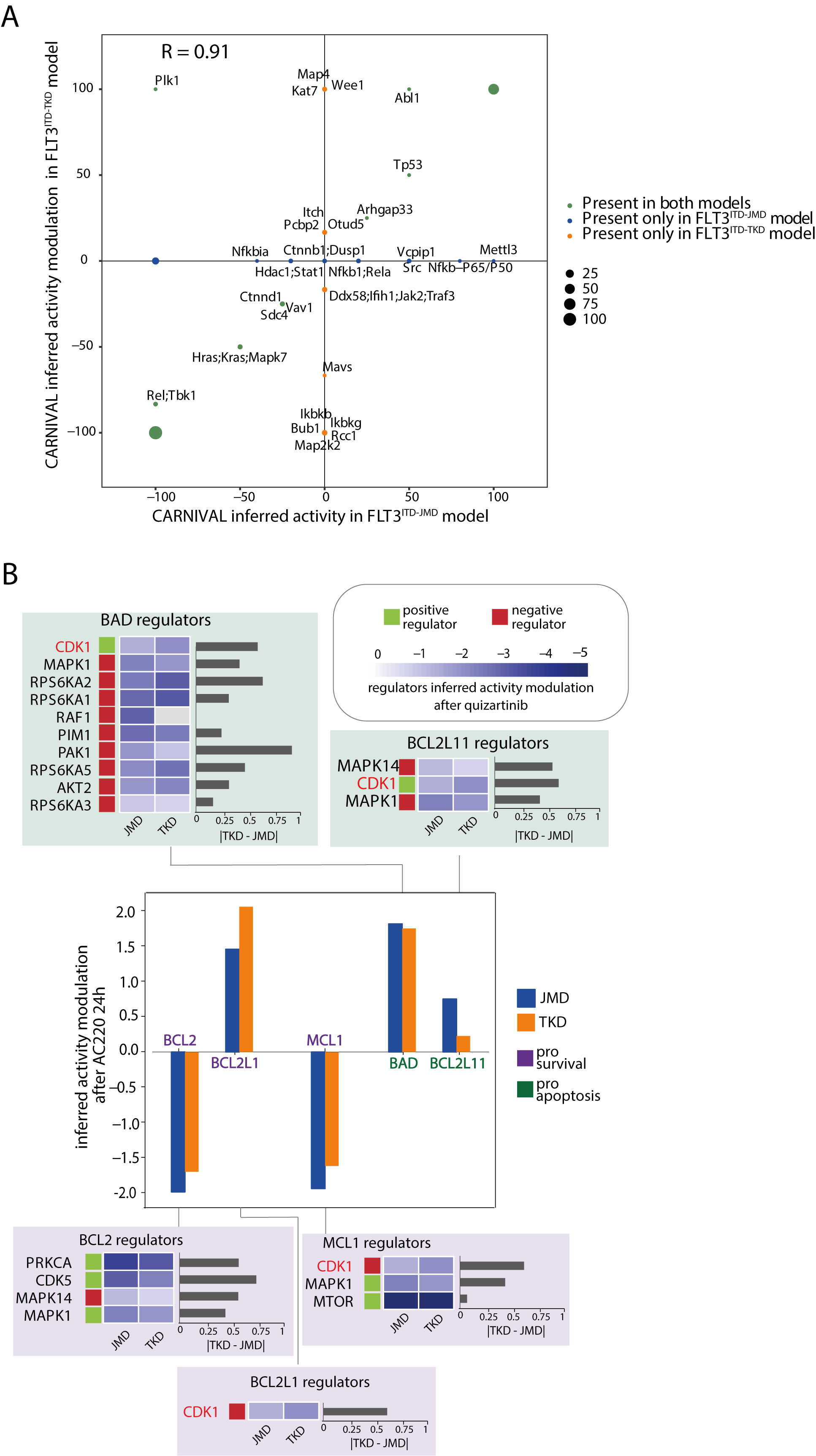

Supplement: Supplementary file 10 — Figure S9 [file 41375_2022_1785_MOESM10_ESM.tif]

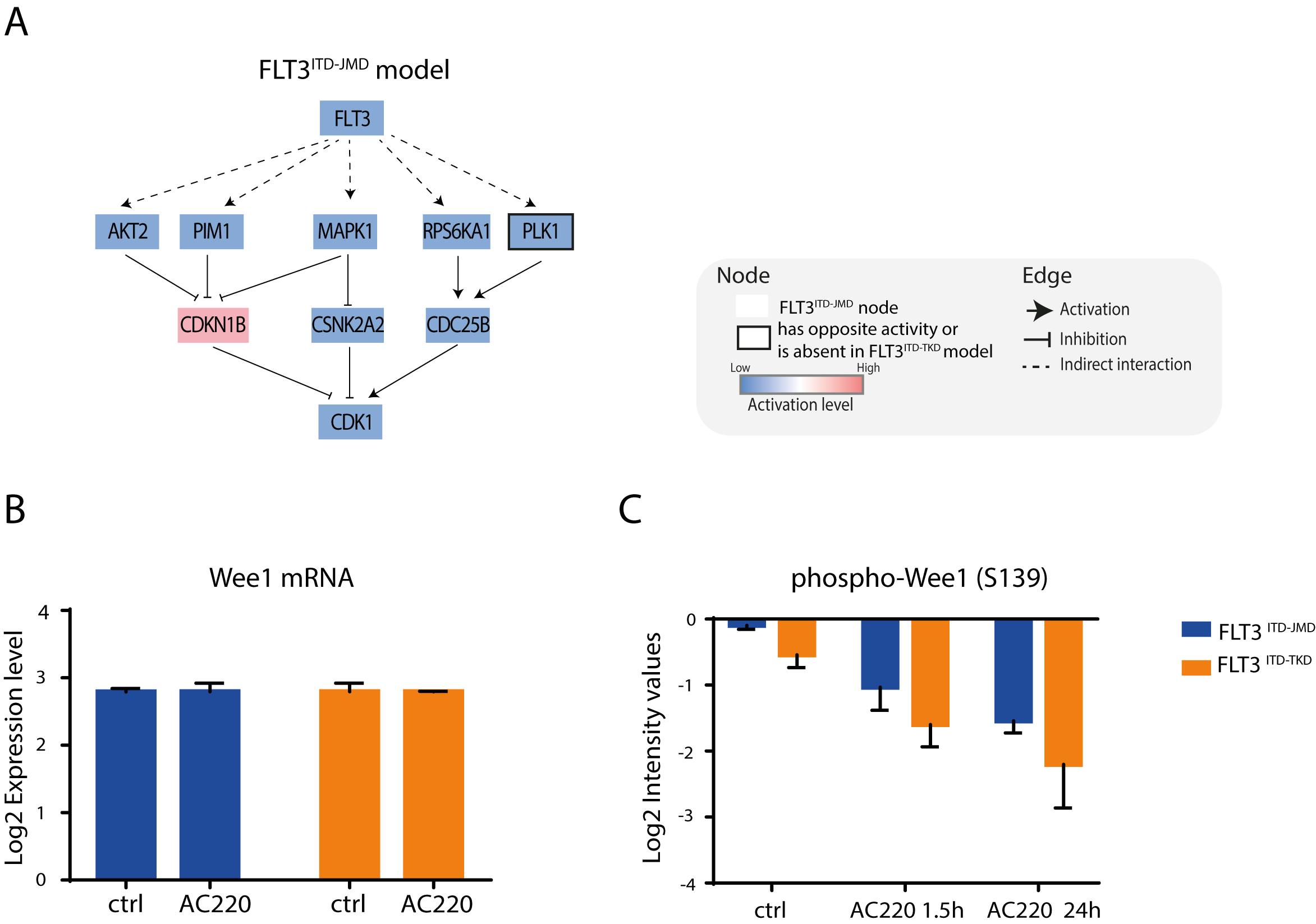

Supplement: Supplementary file 11 — Figure S10 [file 41375_2022_1785_MOESM11_ESM.tif]

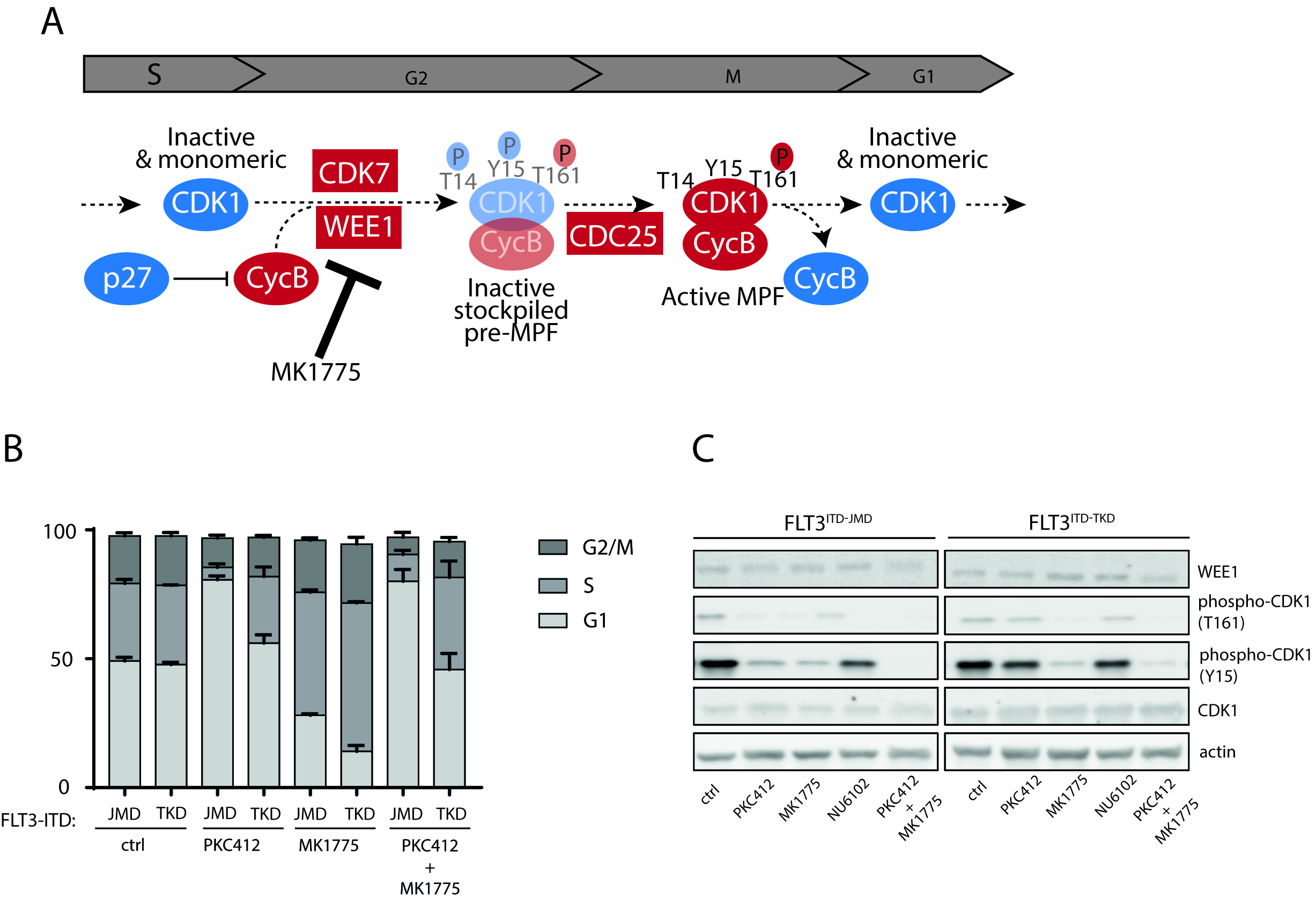

Supplement: Supplementary file 12 — Figure S11 [file 41375_2022_1785_MOESM12_ESM.tif]

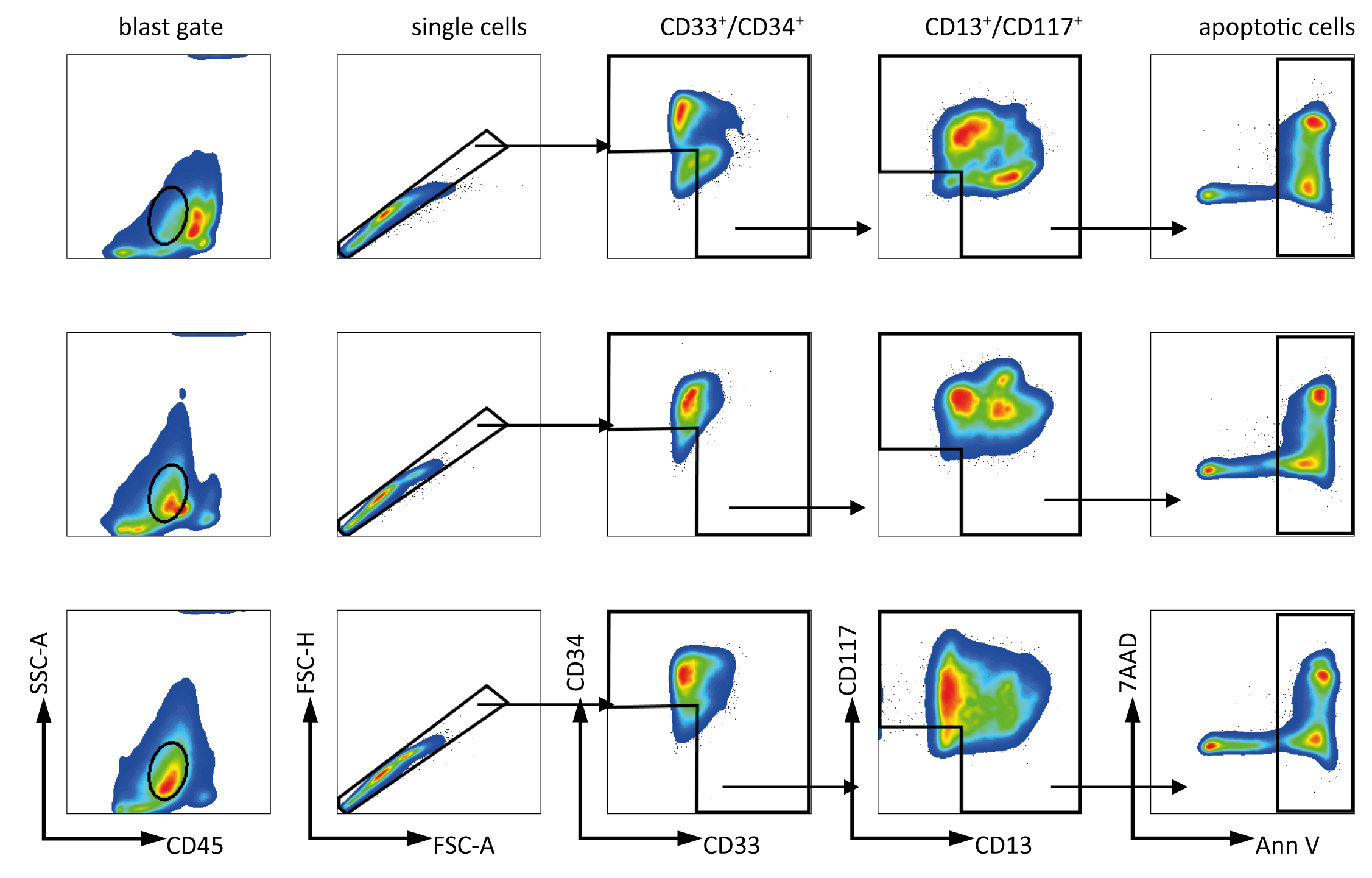

Supplement: Supplementary file 13 — Figure S12 [file 41375_2022_1785_MOESM13_ESM.tif]

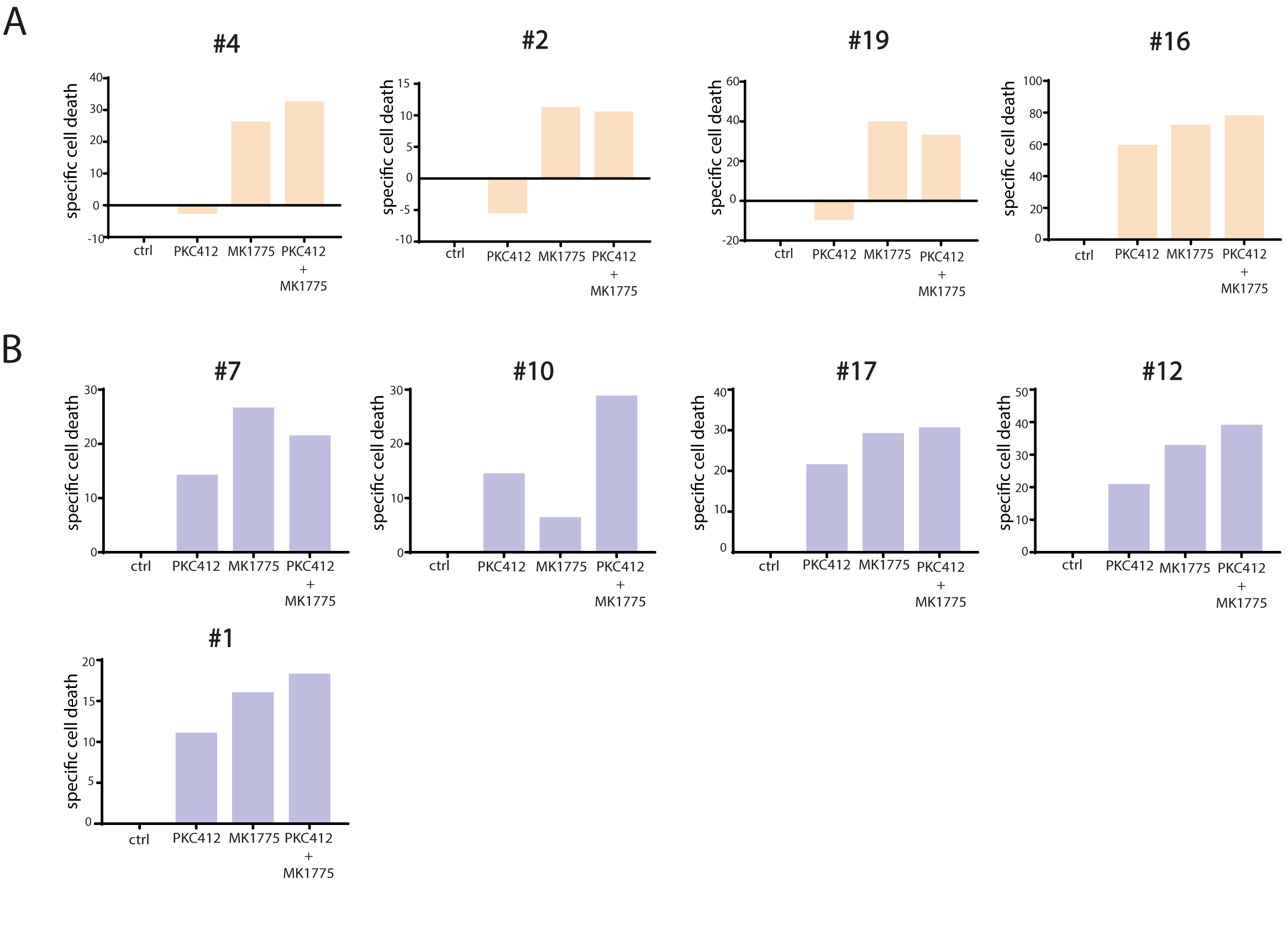

Supplement: Supplementary file 14 — Figure S13 [file 41375_2022_1785_MOESM14_ESM.tif]
